# Mechanisms of liver injury in high fat sugar diet fed mice that lack hepatocyte X-box binding protein 1

**DOI:** 10.1371/journal.pone.0261789

**Published:** 2022-01-14

**Authors:** Xiaoying Liu, Sarah A. Taylor, Kyle D. Gromer, Danny Zhang, Susan C. Hubchak, Brian E. LeCuyer, Takao Iwawaki, Zengdun Shi, Don C. Rockey, Richard M. Green

**Affiliations:** 1 Division of Gastroenterology and Hepatology, Department of Medicine, Northwestern University, Chicago, Illinois, United States of America; 2 Division of Gastroenterology, Hepatology and Nutrition, Department of Pediatrics, Ann & Robert H. Lurie Children’s Hospital of Chicago, Chicago, Illinois, United States of America; 3 Division of Cell Medicine, Department of Life Science, Medical Research Institute, Kanazawa Medical University, Kahoku, Ishikawa, Japan; 4 Digestive Disease Research Center, Medical University of South Carolina, Charleston, South Carolina, United States of America; Texas A&M University, UNITED STATES

## Abstract

Nonalcoholic fatty liver disease (NAFLD) is one of the most common causes of liver diseases in the United States and can progress to cirrhosis, end-stage liver disease and need for liver transplantation. There are limited therapies for NAFLD, in part, due to incomplete understanding of the disease pathogenesis, which involves different cell populations in the liver. Endoplasmic reticulum stress and its adaptative unfolded protein response (UPR) signaling pathway have been implicated in the progression from simple hepatic steatosis to nonalcoholic steatohepatitis (NASH). We have previously shown that mice lacking the UPR protein X-box binding protein 1 (XBP1) in the liver demonstrated enhanced liver injury and fibrosis in a high fat sugar (HFS) dietary model of NAFLD. In this study, to better understand the role of liver XBP1 in the pathobiology of NAFLD, we fed hepatocyte XBP1 deficient mice a HFS diet or chow and investigated UPR and other cell signaling pathways in hepatocytes, hepatic stellate cells and immune cells. We demonstrate that loss of XBP1 in hepatocytes increased inflammatory pathway expression and altered expression of the UPR signaling in hepatocytes and was associated with enhanced hepatic stellate cell activation after HFS feeding. We believe that a better understanding of liver cell-specific signaling in the pathogenesis of NASH may allow us to identify new therapeutic targets.

## Introduction

Nonalcoholic fatty liver disease is the most common cause of abnormal liver function tests in the United States and the progressive form termed nonalcoholic steatohepatitis (NASH) will soon be the leading indication for liver transplantation. There are currently no FDA-approved medical therapies for NASH, in part because the pathogenesis remains poorly understood. NASH is highly correlated with the metabolic syndrome characterized by factors including obesity, insulin resistance or diabetes and dyslipidemia. The metabolic syndrome can cause hepatic steatosis with a resultant increase of endoplasmic reticulum (ER) stress, characterized by an excess of cellular misfolded and unfolded proteins. The unfolded protein response (UPR) is a protective response to ER stress consisting of three pathways: inositol requiring enzyme 1α (IRE1α)/X-box binding protein 1 (XBP1), PKR-like ER kinase (PERK) and activating transcription factor 6 (ATF6), which serve to return cellular homeostasis [1]. Prolonged and severe ER stress that cannot be resolved induces apoptosis.

The UPR has been implicated in the pathogenesis of several liver diseases including viral hepatitis, nonalcoholic and alcoholic fatty liver disease, cholestasis, ischemia reperfusion injury and genetic liver diseases [2]. More specifically, inadequate activation and dysregulation of the hepatic XBP1 pathway has been implicated in NASH [3] and we have previously demonstrated that when fed a high fat sugar (HFS) diet, mice lacking hepatic XBP1 develop increased liver injury and fibrosis compared to control XBP1 flox mice [4]. Although it is well recognized that the pathogenesis of NASH involves both hepatocytes and nonparenchymal cells, cell-specific differences of UPR signaling in the pathogenesis of NASH remain incompletely understood.

In this study, we have fed a HFS diet or regular chow diet to liver-specific XBP1 knockout and control mice to determine the role of the UPR and its associated cellular signaling in the pathogenesis of NASH. We isolated hepatocytes, hepatic stellate cells, macrophages and leukocytes to determine the cell-specific pathways responsible for the development of hepatitis and progressive liver disease. Better understanding this cell signaling will allow us to identify molecular targets that hold promise for this very common form of liver disease with a significant morbidity and mortality.

## Materials and methods

### Animals

Liver-specific XBP1 knockout (*Xbp1*^LKO^) and *Xbp1*^fl/fl^ control mice in a C57BL/6 background strain as previously described [4] were housed on a 14-h light, 10-h dark cycle with free access to food and water. Adult male *Xbp1*^fl/fl^ and *Xbp1*^LKO^ mice were fed either standard chow diet with water or a high fat sugar (HFS) diet [AIN-76 Western Diet (Test Diet) supplemented with drinking water containing 42 g/l of 55% fructose/45% glucose by weight] for up to 6 weeks. IRE1α-flox (ERN1-flox) mice on a C57BL/6/129 mixed background strain developed by Dr. Takao Iwawaki [5] were purchased from Riken, JN (RBRC05515) and a colony was established. Adult male IRE1α-flox mice were injected intravenously with either AAV8-TBG-Cre or AAV8-TBG-eGFP (5x10^10^ vg/mouse, Penn Vector Core) to obtain *Ire1α*^LKO^ or *Ire1α*^fl/fl^ mice, respectively. After 2 weeks, the mice were fed either the HFS diet or chow for 5 weeks. The mice were fasted for 4 hours prior to carbon dioxide euthanasia, blood was obtained using cardiac puncture, the liver was removed and rinsed with ice-cold saline, sectioned and snap-frozen in liquid nitrogen. These studies were carried out in strict accordance with the recommendations in the Guide for the Care and Use of Laboratory Animals of the National Institutes of Health. All protocols and procedures were approved by the Northwestern University Institutional Animal Care and Use Committee (Protocol number #IS00011425).

### Histology

Specimens were fixed in 10% neutral buffered formalin, paraffin-embedded and sectioned. Hematoxylin and eosin (H&E) and cytokeratin 19 (CK19) staining was performed by the Northwestern University Mouse Histology and Phenotyping Laboratory. All histology was assessed by investigators blinded to the treatment groups.

### Biochemical analysis

Serum alanine aminotransferase (ALT) was measured by using a spectrophotometric assay according to the manufacturer’s protocol (Teco Diagnostics, Anaheim, CA). Hepatic triglyceride and cholesterol were determined as described previously [4].

### Cell isolation

Primary hepatocytes were isolated as described [6] with modifications. Isolated hepatocytes were further purified with density centrifugation with Percoll (Sigma, St. Louis, MO). Cell viability was assessed with Trypan Blue. Hepatic stellate cells were isolated as previously described [7] with modifications. Briefly, mouse livers were subjected to a series of enzymatic perfusions (Pronase:1mg/ml; Roche); Collagenase type I (0.12mg/ml, Worthington). The digested livers were incubated in the solution containing Pronase (0.12mg/ml, Roche) and DNase (0.03mg/ml, Sigma) at 37°C with gently shaking for 15 minutes. The hepatic stellate cells were purified with Accudenz gradient (final concentration:10.5%, Accurate Chemical & Scientific Co., Westbury, NY) centrifugation at 4,000 rpm for 25 minutes. The purified hepatic stellate cells were washed and examined for purity as measured by V-A droplet autofluorescence under microscope (purity ≥ 95%).

### Quantitative PCR and RNA-Seq with pathway analysis

Total RNA was extracted from freshly isolated hepatocytes and hepatic stellate cells using Trizol according to the manufacturer’s protocol (Invitrogen Life Technologies, Carlsbad, CA) and cDNA was made with qScript cDNA synthesis kit (Quanta Bioscience, Gaithersburg, MD). Quantitative PCR (qPCR) was performed as described previously [4]. All primers were synthesized by Sigma (St. Louis, MO).

The stranded mRNA-seq was conducted in the Northwestern University NUSeq Core Facility. Briefly, total RNA samples from isolated hepatocytes were checked for quality using RINs generated from Agilent Bioanalyzer 2100. RNA quantity was determined with Qubit fluorometer. The Illumina TruSeq Stranded mRNA Library Preparation Kit was used to prepare sequencing libraries from 1 μg of high-quality RNA samples (RIN>7). The Kit procedure was performed without modifications. This procedure includes mRNA purification and fragmentation, cDNA synthesis, 3’ end adenylation, Illumina adapter ligation, library PCR amplification and validation. lllumina HiSeq 4000 sequencer was used to sequence the libraries with the production of single-end, 50 bp reads at the depth of 20–25 M reads per sample. The RNA-Seq data, in fastq format, was quantified using kallisto. Quantification data was imported using tximport and differential gene expression was performed using DESeq2. The cutoff for determining significantly differentially expressed genes was an FDR-adjusted p-value less than 0.05. Gene set enrichment analysis (GSEA) was conducted using fgsea. The C8 collection of gene sets from Molecular Signatures Database (MSigDB) was used to identify cell type specific pathway enrichment and the Hallmark collection of gene sets from MSigDB was used to identify enriched pathways in well-defined biological processes. The RNA-Seq data of whole liver RNA has been previously published and deposited to Gene Expression Omnibus database (GSE64824) [4]. Differentially expressed immune genes were defined by one investigator (S.A.T) based on previously known function.

### Western blotting

Protein extraction was made from freshly isolated hepatocytes and hepatic stellate cells using T-Per (Thermo Scientific, Waltham, MA) or RIPA protein extraction reagent, respectively, with protease inhibitors cocktail (MilliporeSigma, Burlington, MA) and phosphatase inhibitors (Thermo Scientific, Waltham, MA). Protein quantification and immunoblotting were performed as described previously [4].

### Flow cytometry analysis of immune cells

Tissue digestion and cell isolation for flow cytometry was performed as previously described [8]. Briefly, cardioperfusion was performed with PBS and whole liver tissue was finely cut into small pieces and added to a c-tube with 2.5 ml digestion buffer per tube composed of DNase I (Sigma), Liberase TL (Sigma), and HBSS (Gibco). Further tissue digestion was achieved using the Miltenyi Biotec gentle MACS Dissociator and incubation with shaking at 37°C for 30 minutes. The homogenate was maintained at 4°C and strained through a 40 μm filter into a 50 mL conical tube. Pharm Lyse was used to lyse remaining red blood cells. Samples were spun at 350 rcf for 7 min (4°C) and cells were washed prior to performing cell counts.

Cells were re-suspended at a concentration of 1.0 x 10^7^ cells per 90 μl MACS buffer and 10 μl of mouse CD45 MicroBeads (Miltenyi Biotec) per 1.0 x 10^7^ cells for CD45 enrichment. The solution was incubated for 15 minutes at 4°C with intermittent gentle mixing before magnetic separation with MS columns on the MACS Separator per standard protocol (Miltenyi Biotec). Single cell suspensions from each individual mouse were stained with a panel of 12 antibodies to detect cell viability and expression of the following cell surface markers: CD45, Siglec-F, Ly6g, CD3, CD19, CD11b, CD64, Ly6c, and CD11c. Flow cytometry was performed on the BD FACSAria cell sorter at the Robert H. Lurie Comprehensive Cancer Center Flow Cytometry Core Facility and data was analyzed using FlowJo software.

### Statistics

Data are shown as means ± SEM using PRISM 9 software. Comparisons were performed using a two-tailed Student’s t-test or one-way ANOVA with Bonferroni post hoc test. Statistical significance was defined as P-values of less than 0.05.

## Results

**Fig 1:**
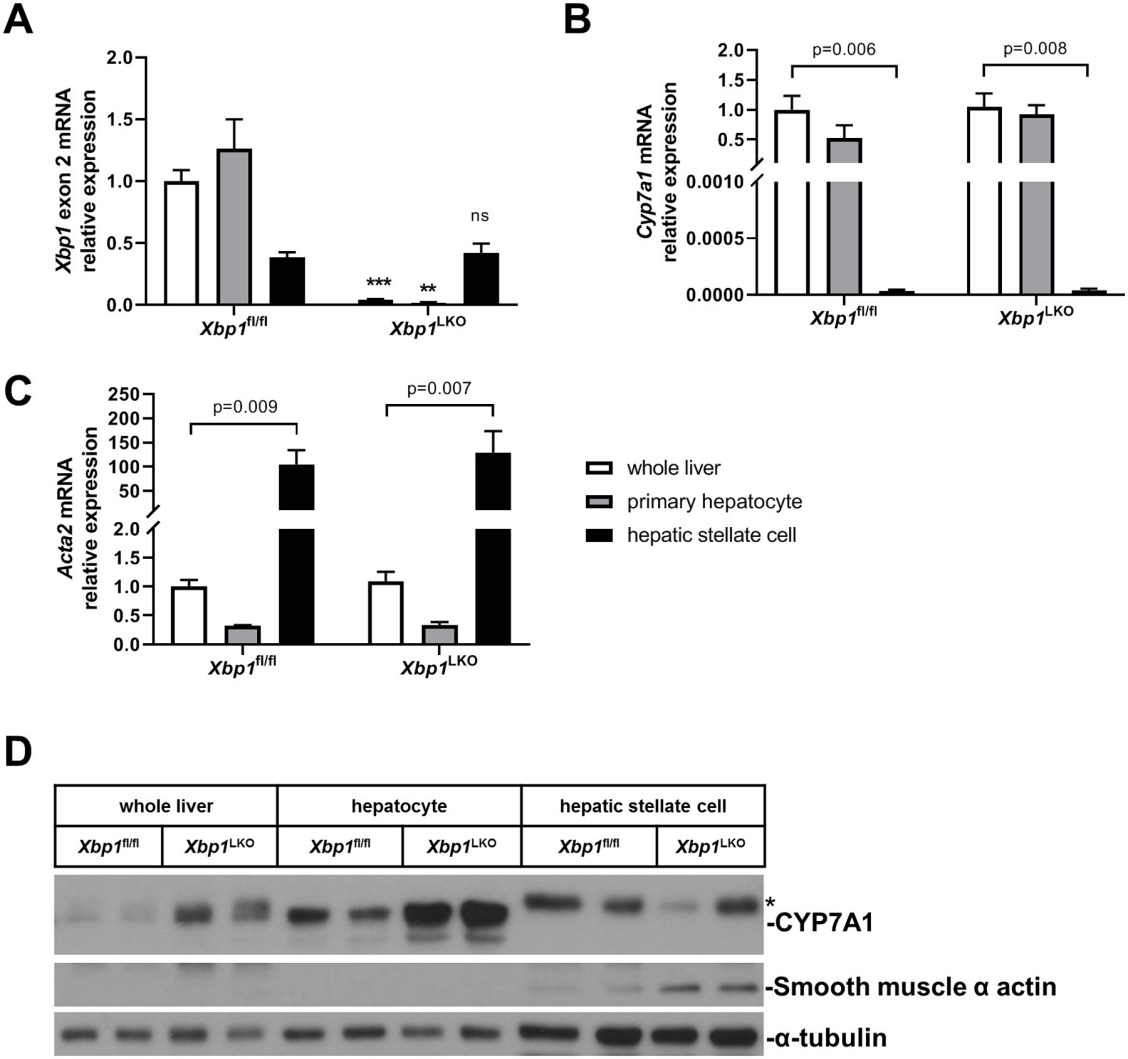
Expression of hepatocyte and hepatic stellate cell-specific markers in cells isolated from *Xbp1*^LKO^ and *Xbp1*^fl/fl^ mice. Hepatocytes and hepatic stellate cells were isolated from chow fed *Xbp1*^LKO^ and *Xbp1*^fl/fl^ mice (n = 3–4 in each group). qPCR was used to measure isolated cells and whole liver gene expression of (A) *Xbp1* exon 2, (B) *Cyp7a1*, and (C) *Acta2*. White box, whole liver; Grey box, primary hepatocyte; black box, hepatic stellate cell. **p<0.01 or ***p<0.001, ns, not significant compared to *Xbp1*^fl/fl^ mice. (D) Protein expression of CYP7A1 and smooth muscle α actin was examined by western blotting with α-tubulin as a loading control. The asterisk (*) indicates non-specific band.

In order to better understand the role of hepatic XBP1 in NASH, we utilized the HFS dietary model of NASH to investigate the cell-specific signaling pathways in hepatocytes and non-parenchymal cells. We initially confirmed that deletion of XBP1 was hepatocyte-specific and complete, and confirmed the purity of isolated hepatocytes and hepatic stellate cells (Fig 1A). *Xbp1* exon 2 expression was essentially absent in primary hepatocytes isolated from albumin promoter-Cre *Xbp1*^LKO^ mice, consistent with their predicted genotype (p = 0.006). In contrast, *Xbp1* exon 2 expression in hepatic stellate cells was normal. The enrichment and purity of isolated hepatocytes and hepatic stellate cells were further confirmed by gene and protein expression of hepatocyte-specific marker (*Cyp7a1*) and hepatic stellate cell-specific marker (smooth muscle α actin, also *Acta2*) (Fig 1B–1D).

### RNA-Seq analysis of hepatocytes isolated from *Xbp1*^LKO^ and *Xbp1*^fl/fl^ mice fed either chow or HFS diet

To define the hepatocyte cell signaling responsible for the pathogenesis of steatohepatitis in *Xbp1*^LKO^ and *Xbp1*^fl/fl^ mice, we fed both genotypes either chow or HFS diet for up to 6 weeks. S1 Fig demonstrated the top 20 cell type signature specific gene pathways that were most differentially expressed in hepatocytes isolated from *Xbp1*^LKO^ and *Xbp1*^fl/fl^ mice and in whole liver from similarly treated mice [4] (GSE64824). Whole liver RNA-Seq analysis demonstrates enhanced expression of stellate cell and bile duct pathways in chow-fed *Xbp1*^LKO^ mice and significantly reduced expression of multiple liver-specific pathways compared to *Xbp1*^fl/fl^ mice (S1A Fig). In contrast, there were no increases in expression of hepatic non-parenchymal cell pathways in hepatocytes isolated from chow-fed *Xbp1*^LKO^ mice compared to *Xbp1*^fl/fl^ hepatocytes, while expression of multiple liver-specific pathways was again reduced (S1B Fig). S1C and S1D Fig demonstrate similar analysis of whole liver and isolated hepatocytes from mice fed a HFS diet. Following HFS dietary feeding, whole liver pathway analysis revealed enhanced expression of stellate cell, immune cell (e.g. macrophages, kupffer cells) and bile duct cell signatures, with reduced expression of hepatocyte-specific pathways in *Xbp1*^LKO^ mice compared to *Xbp1*^fl/fl^ mice (S1C Fig). In contrast, hepatocytes from HFS-fed *Xbp1*^LKO^ mice did not have increased expression for non-parenchymal liver cell signatures (S1D Fig). These data not only further confirmed the purity of the hepatocyte preparations, but also suggested that significant hepatocyte cell signaling may be obscured by non-parenchymal cell signaling in whole liver gene or protein expression analyses. Of note, 3 gene sets containing curated cluster markers for bile duct cells were enriched in HFS-fed *Xbp1*^LKO^ mice livers compared to HFS-fed *Xbp1*^fl/fl^ mice livers. This finding was further confirmed by CK19 staining showing increased ductular reaction in HFS-fed *Xbp1*^LKO^ mice livers compared to HFS-fed *Xbp1*^fl/fl^ mice livers (S2 Fig). There was no significant difference in CK19 staining between chow-fed *Xbp1*^LKO^ mice and *Xbp1*^fl/fl^ mice livers.

**Fig 2:**
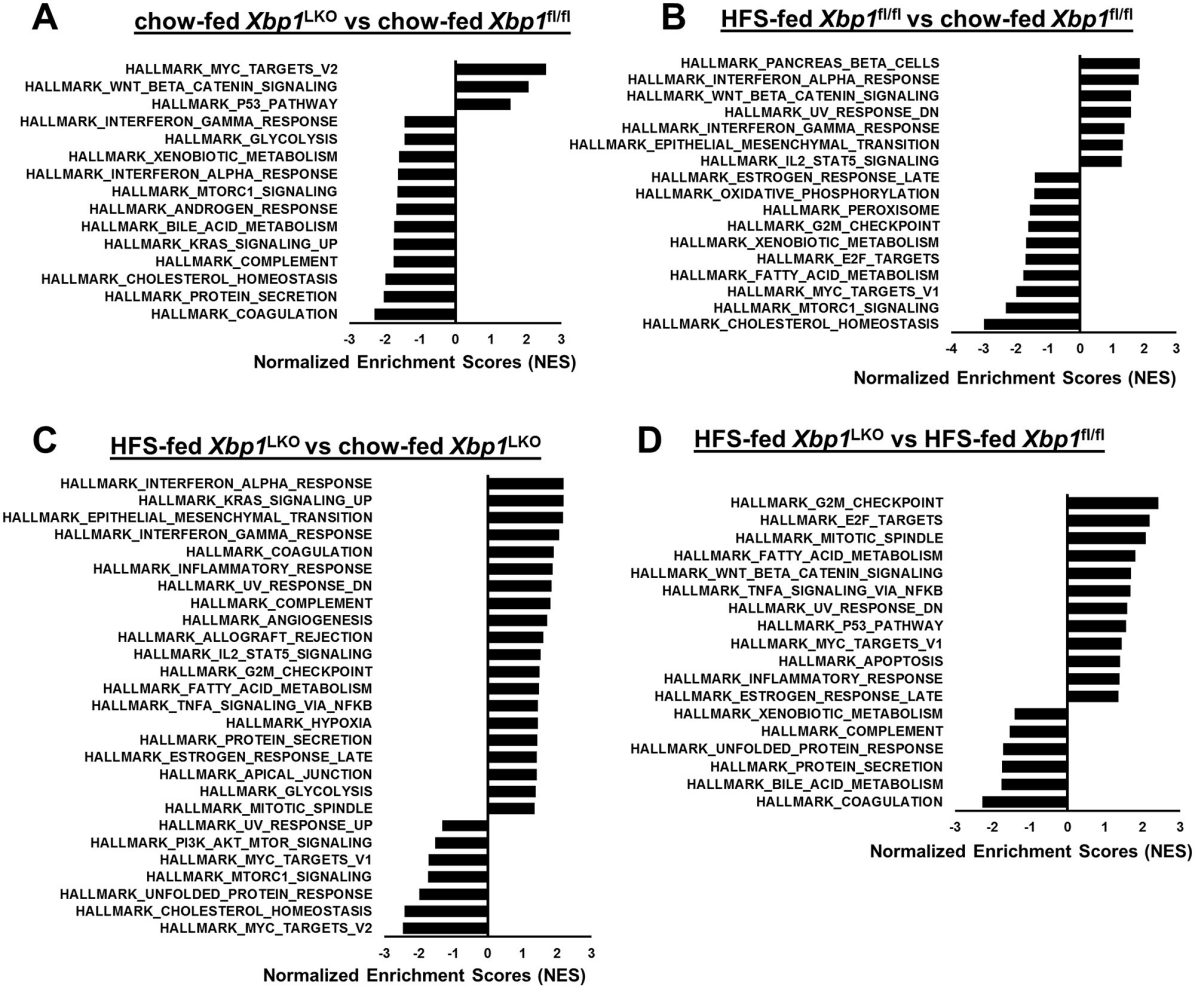
Hallmark pathway analysis comparing gene expression in hepatocytes isolated from *Xbp1*^LKO^ and *Xbp1*^fl/fl^ mice fed either chow or HFS diet. RNA-Seq and Hallmark pathway analysis was performed on: (A) *Xbp1*^LKO^ mice fed chow compared to *Xbp1*^fl/fl^ mice fed chow, (B) *Xbp1*^fl/fl^ mice fed HFS diet compared to *Xbp1*^fl/fl^ mice fed chow. (C) *Xbp1*^LKO^ mice fed HFS diet compared *Xbp1*^LKO^ mice fed chow, and (D) *Xbp1*^LKO^ mice fed HFS diet compared to *Xbp1*^*f*l/fl^ mice fed HFS diet. n = 3 in chow-fed groups and n = 3 (pooled samples of 2 animals) in HFS-fed groups. Significantly enriched pathways with p-adj<0.05 were shown.

We subsequently used Hallmark pathway analysis of hepatocyte RNA-Seq to determine changes in hepatocyte gene expression patterns in *Xbp1*^LKO^ and *Xbp1*^fl/fl^ mice fed either diet. Fig 2A demonstrates that hepatocytes isolated from chow-fed *Xbp1*^LKO^ mice had lower expression of Interferon_Alpha_Response and Interferon_Gamma_Response pathways than hepatocytes isolated from chow-fed *Xbp1*^fl/fl^ mice. However, there were no differences in the expression of any other inflammatory or cytokine pathways. When *Xbp1*^fl/fl^ mice were fed a HFS diet, hepatocyte expression of Interferon_Alpha_Response, Interferon_Gamma_Response and IL2_STAT5_Signaling pathways increased, while other inflammatory pathways remained unaffected (Fig 2B). In contrast, hepatocytes from *Xbp1*^LKO^ mice fed the HFS diet had increased expression of several additional liver injury or inflammation associated pathways, including Inflammatory_Response, Complement and TNFA_ Signaling_Via_NFκB pathways compared to HFS-fed *Xbp1*^fl/fl^ mice (Fig 2C). There was also a trend towards upregulation of apoptosis pathways in HFS-fed *Xbp1*^LKO^ mice compared to chow-fed *Xbp1*^LKO^ mice which did not reach statistical significance (p-adj = 0.07). While hepatocytes from chow-fed *Xbp1*^LKO^ and *Xbp1*^fl/fl^ mice differed only in interferon pathway expression, Fig 2D demonstrates that in response to HFS dietary feeding, hepatocytes from *Xbp1*^LKO^ mice had greater expression of TNFA_ Signaling_Via_NFκB, Apoptosis and Inflammatory_Response pathways, but no differences in interferon pathways compared to hepatocytes from HFS-fed *Xbp1*^fl/fl^ mice.

Consistent with the known mechanisms for regulation of lipid metabolism by the IRE1α/XBP1 pathway [9, 10], hepatocyte Bile_Acid_Metabolism and Cholesterol_Homeostasis pathway expression was lower in chow-fed *Xbp1*^LKO^ compared to chow-fed *Xbp1*^fl/fl^ mice (Fig 2A). When fed HFS diet, Bile_Acid_Metabolism but not Cholesterol_Homeostasis pathway remained lower in *Xbp1*^LKO^ hepatocytes compared to *Xbp1*^fl/fl^ hepatocytes, while pathway expression for Cholesterol_Homeostasis was lower in both genotypes after HFS feeding (Fig 2B–2D). There were also no differences in Fatty_Acid_Metabolism pathways in the hepatocytes of two genotypes of mice fed chow, while in contrast, when fed the HFS diet there were genotype-specific differences in fatty acid metabolism pathways. *Xbp1*^fl/fl^ mice had reduced expression, whereas *Xbp1*^LKO^ mice had increased expression of Fatty_Acid_Metabolism pathway after HFS feeding (Fig 2B and 2C).

### Hepatocyte UPR and apoptotic signaling in *Xbp1*^LKO^ and *Xbp1*^fl/fl^ mice fed either chow or HFS diet

**Fig 3:**
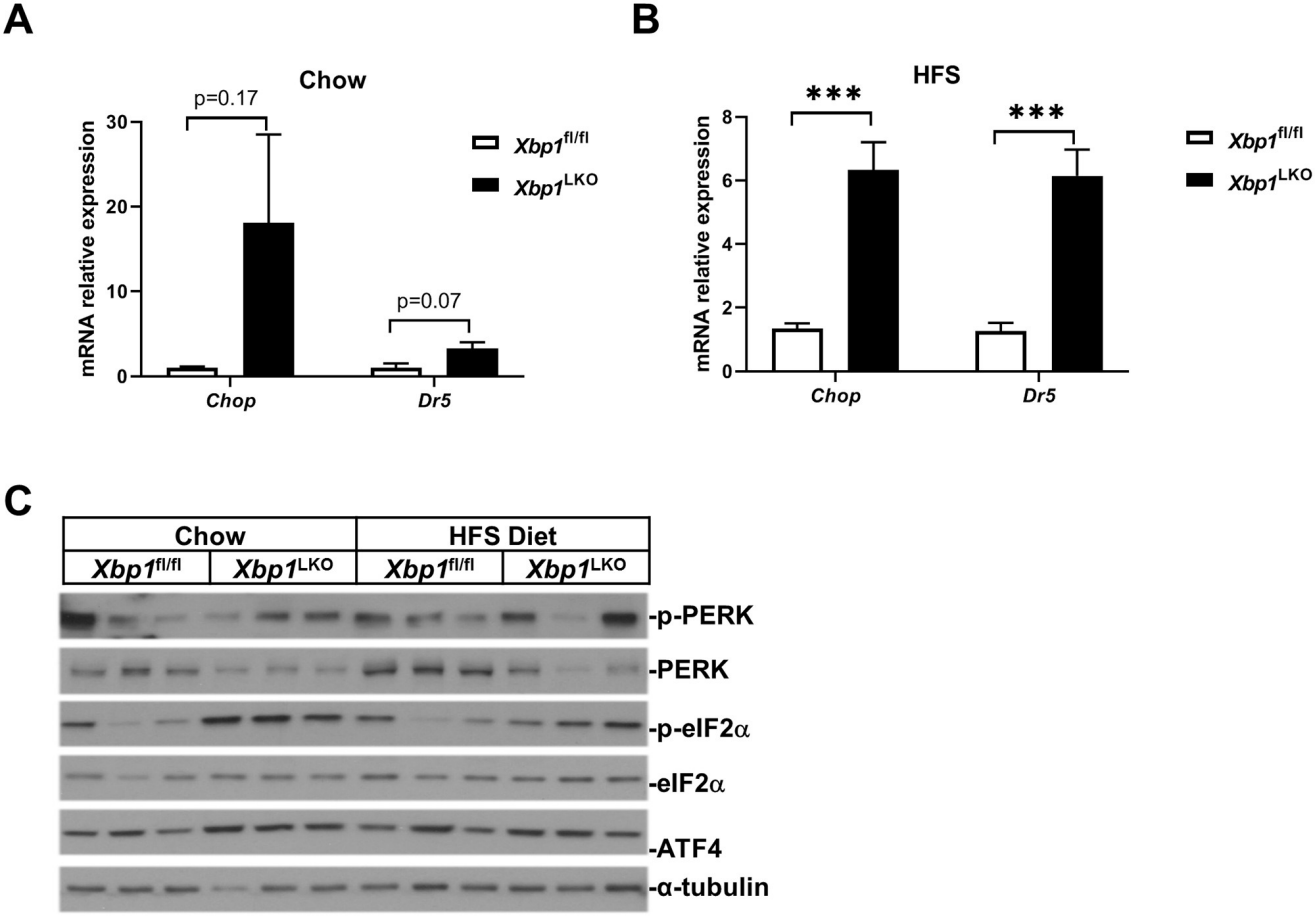
Hepatocyte p-eIF2α/ATF4/CHOP pathway expression was increased in *Xbp1*^LKO^ compared to *Xbp1*^fl/fl^ mice. Hepatocytes were isolated from *Xbp1*^fl/fl^ and *Xbp1*^LKO^ mice fed chow (n = 3 of each genotype) or HFS diet (n = 6 of each genotype). Gene and protein expression were examined by qPCR and western blotting. (A) The hepatocyte gene expression of *Chop* and *Dr5* was not significantly different in chow-fed *Xbp1*^fl/fl^ and *Xbp1*^LKO^ mice. (B) The hepatocyte gene expression of *Chop* and *Dr5* was higher in HFS-fed *Xbp1*^LKO^ mice compared to HFS-fed *Xbp1*^fl/fl^ mice. ***p<0.001 compared to *Xbp1*^fl/fl^ mice. (C) The protein expression of p-PERK, PERK, p-eIF2α, eIF2α and ATF4 was examined by western blotting with α-tubulin as a loading control. Each lane in HFS-fed samples represents pool lysates from 2 mice. The expression of p-eIF2α and ATF4 was increased in either chow fed HFS fed *Xbp1*^LKO^ mice compared to *Xbp1*^fl/fl^ mice.

We have previously shown that liver C/EBP homologous protein (*Chop*) gene expression is significantly higher in *Xbp1*^LKO^ mice compared to *Xbp1*^fl/fl^ mice [4], therefore we examined *Chop* gene expression in hepatocytes isolated from chow-fed or HFS diet-fed *Xbp1*^LKO^ and *Xbp1*^fl/fl^ mice. The gene expression of *Chop* and its downstream target death-receptor 5 (*Dr5*) was not significantly different in chow-fed *Xbp1*^LKO^ and *Xbp1*^fl/fl^ hepatocytes (Fig 3A), but was significantly higher in HFS-fed *Xbp1*^LKO^ hepatocytes compared to HFS-fed *Xbp1*^fl/fl^ hepatocytes (Fig 3B, p<0.001).

CHOP can be activated via the PERK/eukaryotic translation initiation factor 2α/activating transcription factor 4 (PERK/eIF2α/ATF4) pathway. Protein expression of PERK, but not phosphorylated PERK (p-PERK), was lower in *Xbp1*^LKO^ hepatocytes compared to *Xbp1*^fl/fl^ hepatocytes on either diet (Fig 3C), although gene expression of hepatocyte PERK (*Eif2ak3*) was not different between the two genotypes (Table 1). PERK activates its downstream target eIF2α by phosphorylation. The expression of p-eIF2α was increased in *Xbp1*^LKO^ hepatocytes compared to *Xbp1*^fl/fl^ hepatocytes on either diet while total eIF2α was similar in both genotypes (Fig 3C). Protein expression of ATF4 was also elevated in *Xbp1*^LKO^ hepatocytes compared to *Xbp1*^fl/fl^ hepatocytes. In addition to PERK, eIF2α can be phosphorylated by 3 other kinases in the integrated stress response (ISR) pathway: heme-regulated eIF2α kinase (HRI, *Eif2ak1*), double-stranded RNA-dependent protein kinase (PKR, *Eif2ak2*), and general control non-derepressible 2 (GCN2, *Eif2ak4*). The gene expression of these eIF2α kinases were unaffected by genotype or diet, except *Eif2ak1* gene expression was slightly decreased in *Xbp1*^LKO^ hepatocytes after HFS feeding (Table 1). Constitutive repressor of eIF2alpha phosphorylation (CReP) is a member of the ISR and is involved in the de-phosphorylation of p-eIF2α. CReP is also regulated by IRE1-dependent decay of messenger RNAs (RIDD) [11]. Hepatocytes from chow or HFS-fed *Xbp1*^LKO^ mice compared to *Xbp1*^fl/fl^ mice had a 40% reduction in CReP (*Ppp1r15b*) gene expression (Table 1) consistent with increased p-eIF2α protein expression. Hepatocytes from both chow and HFS diet fed *Xbp1*^LKO^ mice also had marked reduction of gene expression of other RIDD targets (*Bloc1s1*, *Angptl3*, *Dgat2*) (Table 1).

**Table 1:** RNA-Seq gene expression of eIF2α kinases and RIDD targets in *Xbp1*^LKO^ and *Xbp1*^fl/fl^ hepatocytes.

Gene Name	chow (FC)	p-adj_chow	HFS Diet (FC)	p-adj_HFS
**eIF2α kinases**				
*Eif2ak1*	0.849	0.268	0.791	0.028
*Eif2ak2*	0.732	0.098	1.031	0.954
*Eif2ak3*	0.959	0.960	0.890	0.888
*Eif2ak4*	0.925	0.840	0.974	0.964
**RIDD targets**				
*Bloc1s1*	0.213	1.78E-10	0.238	1.61E-11
*Angptl3*	0.246	1.08E-12	0.322	2.31E-10
*Ppp1r15b*	0.590	2.42E-05	0.629	3.72E-05
*Dgat2*	0.321	3.08E-08	0.271	2.81E-13

Hepatocyte RNA-seq data was used to compare the gene expression of eIF2α kinases and selected RIDD targets in *Xbp1*^LKO^ and *Xbp1*^fl/fl^ mice fed chow or HFS diet. FC: fold change.

**Fig 4:**
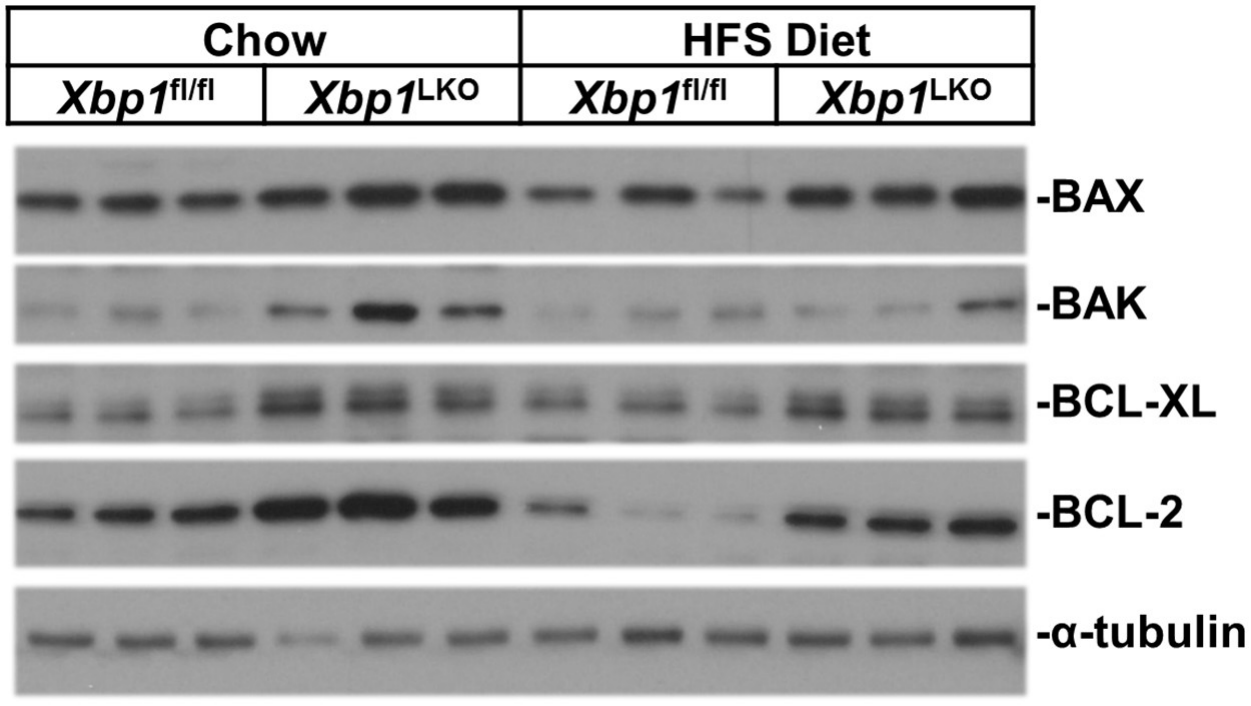
Expression of BCL-2 family proteins in hepatocytes isolated from *Xbp1*^LKO^ and *Xbp1*^fl/fl^ mice fed chow or HFS diet. Hepatocyte protein expression of BAX, BAK, BCL-2 and BCL-XL was greater in chow fed *Xbp1*^LKO^ mice compared to *Xbp1*^fl/fl^ mice. Although protein expression did not increase with HFS diet feeding, the hepatocyte protein expression of BAX, BCL-2 and BCL-XL remained higher in *Xbp1*^LKO^ mice. Each lane in HFS-fed samples represents pool lysates from 2 mice.

Lastly, we determined the effect of XBP1 deficiency on protein expression of proapoptotic proteins regulated by UPR including BAX (induced by CHOP) and other BCL-2 family members in isolated hepatocytes. Fig 4 demonstrates the expression of hepatocyte BCL-2 family proteins in chow and HFS diet fed *Xbp1*^LKO^ and *Xbp1*^fl/fl^ mice. BAX protein expression was significantly higher in *Xbp1*^LKO^ hepatocytes when fed either diet, and BAK was higher in chow fed *Xbp1*^LKO^ hepatocytes. The anti-apoptotic proteins BCL-2 and BCL-XL were also higher in hepatocytes from *Xbp1*^LKO^ mice fed chow or HFS diet compared with *Xbp1*^fl/fl^ mice.

### Hepatic stellate cells were activated in *Xbp1*^LKO^ mice after HFS feeding

**Fig 5:**
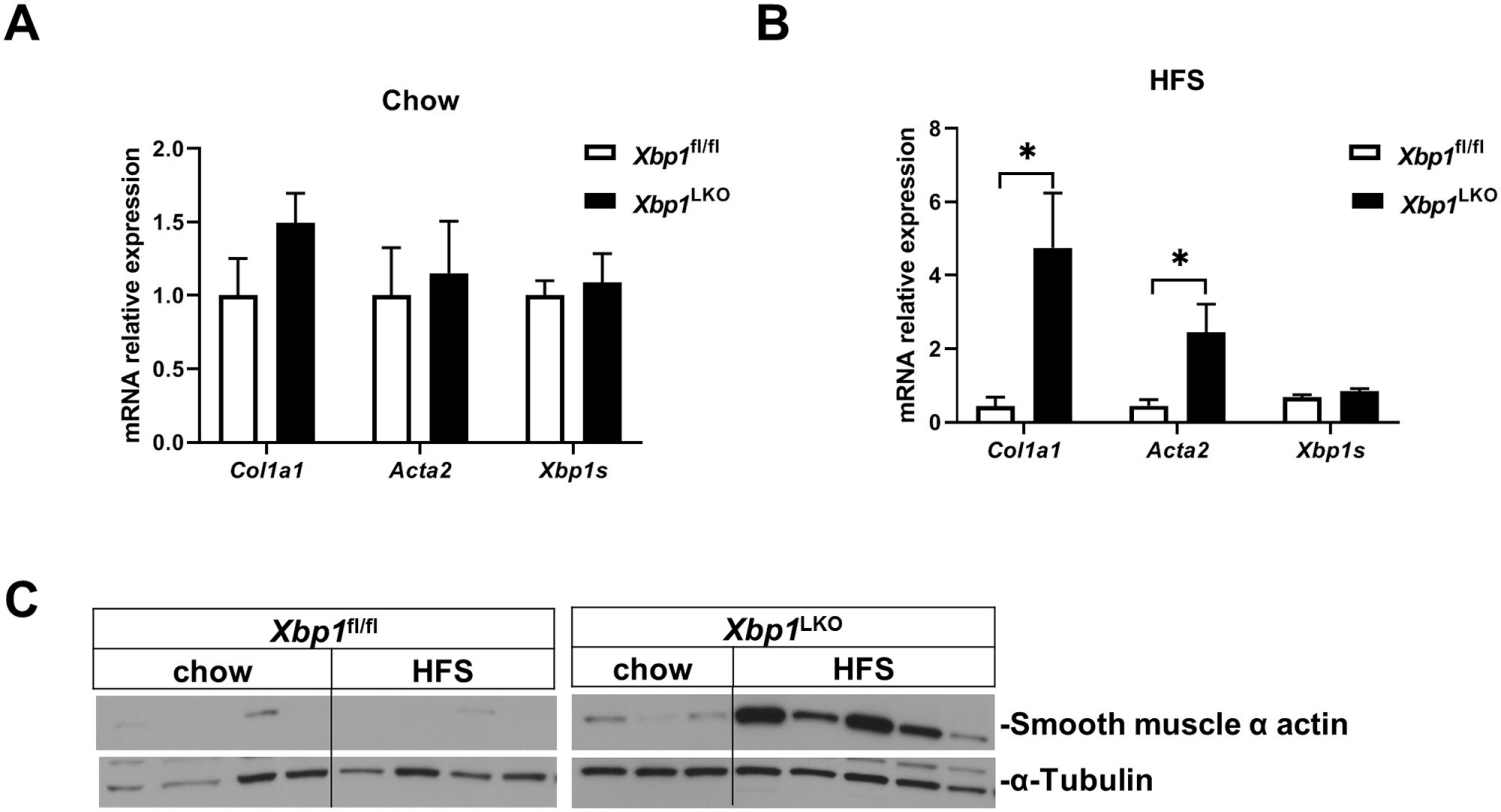
Hepatic stellate cell activation was increased in *Xbp1*^LKO^ mice fed HFS diet. Hepatic stellate cells were isolated from *Xbp1*^fl/fl^ and *Xbp1*^LKO^ mice fed chow (n = 3–4 of each genotype) or HFS diet (n = 4–5 of each genotype). (A) Hepatic stellate cell gene expression of *Col1a1*, *Acta2* and *Xbp1s* was similar between chow fed *Xbp1*^fl/fl^ and *Xbp1*^LKO^ mice. (B) Hepatic stellate cell gene expression of *Col1a1* and *Acta2* was higher in *Xbp1*^LKO^ mice compared to *Xbp1*^fl/fl^ mice after HFS feeding. *Xbp1s* expression remained similar between the two genotypes. *p<0.05 compared to HFS fed *Xbp1*^fl/fl^ mice. (C) Western blotting showed that hepatic stellate cell protein expression of smooth muscle α actin was increased in *Xbp1*^LKO^ mice, but not in *Xbp1*^fl/fl^ mice after HFS feeding.

We isolated hepatic stellate cells from both *Xbp1*^LKO^ and *Xbp1*^fl/fl^ mice fed either chow or HFS diet. Hepatic stellate cell gene expression of *Co1a1* and *Acta2* was similar in chow fed *Xbp1*^LKO^ and *Xbp1*^fl/fl^ mice (Fig 5A). After HFS feeding, hepatic stellate cells isolated from *Xbp1*^LKO^ mice had enhanced gene expression of *Col1a1* and *Acta2*, being 10-fold higher (p = 0.02) and 6-fold higher (p = 0.03) compared to *Xbp1*^fl/fl^ mice, respectively (Fig 5B). The gene expression of the active XBP1 spliced form (*Xbp1s*) was unchanged under chow or HFS diet (Fig 5A and 5B). Western blotting revealed that smooth muscle α actin protein expression was minimal in hepatic stellate cells isolated from chow fed mice or HFS diet fed *Xbp1*^fl/fl^ mice, and was abundantly expressed in hepatic stellate cells isolated from HFS diet fed *Xbp1*^LKO^ mice (Fig 5C).

### XBP1 regulation of the hepatic immune response

**Fig 6:**
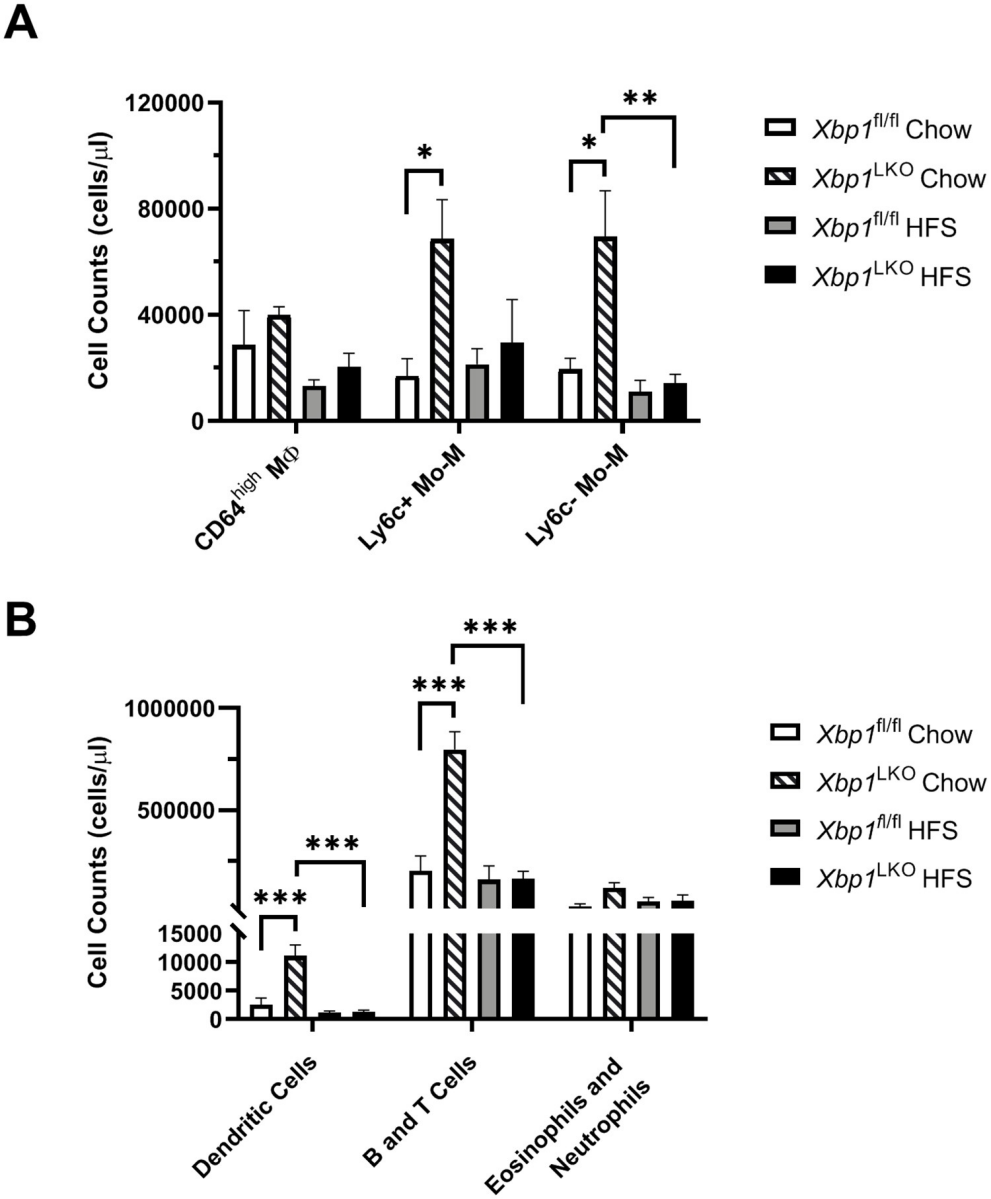
Increased immune cell recruitment in chow fed *Xbp1*^LKO^ mice. *Xbp1*^LKO^ and *Xbp1*^fl/fl^ mice were fed chow or HFS diet and immune cells were characterized using flow cytometry (n = 4 in each group). (A) The number of Ly6c+ and Ly6c- monocyte-derived macrophages (Mo-M) were higher in chow fed *Xbp1*^LKO^ mice compared to chow fed *Xbp*1^fl/fl^ mice. (B) Analysis of other immune cell subsets demonstrates an increase in the number of dendritic cells and adaptive immune cells in chow fed *Xbp1*^LKO^ mice, whereas the number of eosinophils and neutrophils was similar in all groups. No immune cell subsets were significantly different in HFS diet fed *Xbp1*^LKO^ mice compared to *Xbp1*^fl/fl^ mice. *p<0.05; **p<0.01, ***p<0.001.

To better characterize the immune cell subsets responsible for the hepatic inflammatory response, we performed flow cytometry on CD45+ cells from liver tissues of *Xbp1*^LKO^ and *Xbp1*^fl/fl^ mice fed chow or HFS diet (S3 Fig). A range of 1.52 x 10^6^ to 5.28 x 10^7^ total cells were obtained for all samples. Increased numbers of both Ly6c+ and Ly6c- monocyte-derived macrophages in chow-fed *Xbp1*^LKO^ compared to *Xbp1*^fl/fl^ mice (p = 0.038 and 0.010, respectively) were detected using flow cytometry analysis of 3 hepatic macrophage cell subsets (Fig 6A). Chow fed *Xbp1*^LKO^ mice also had more Ly6c- monocyte-derived macrophages than HFS diet fed *Xbp1*^LKO^ mice (p = 0.005). In contrast, the number of CD64^high^ macrophages did not differ between groups. Chow fed *Xbp1*^LKO^ mice also had significantly more dendritic cells and adaptive immune cells (B and T cells) compared to both chow fed *Xbp1*^fl/fl^ and HFS fed *Xbp1*^LKO^ mice (p< 0.001 for all comparisons) (Fig 6B). The numbers of eosinophils and neutrophils were similar among all groups.

We next identified genes known to play a role in immune function that were differentially expressed in whole liver but not hepatocyte RNA-Seq analysis. There were 16 immune genes that were differentially expressed between *Xbp1*^fl/fl^ and *Xbp1*^LKO^ mice fed chow. Of these, 4 genes were only different on chow but not HFS diet, and 12 genes were different on either diet (S4A and S4C Fig). In addition, 87 immune genes were differentially expressed between *Xbp1*^fl/fl^ and *Xbp1*^LKO^ mice fed HFS diet, including 75 genes that were only different on HFS diet, but not chow, and 12 genes that differed on either diet (S4B and S4C Fig). Among these are genes encoding various cytokines and chemokines, genes involved in antigen presentation, interferon response genes, and tumor necrosis factor signaling, consistent with the increased inflammatory response in *Xbp1*^LKO^ mice fed HFS diet [4].

### Deletion of hepatic IRE1α did not alter liver injury in mice fed HFS diet

**Fig 7:**
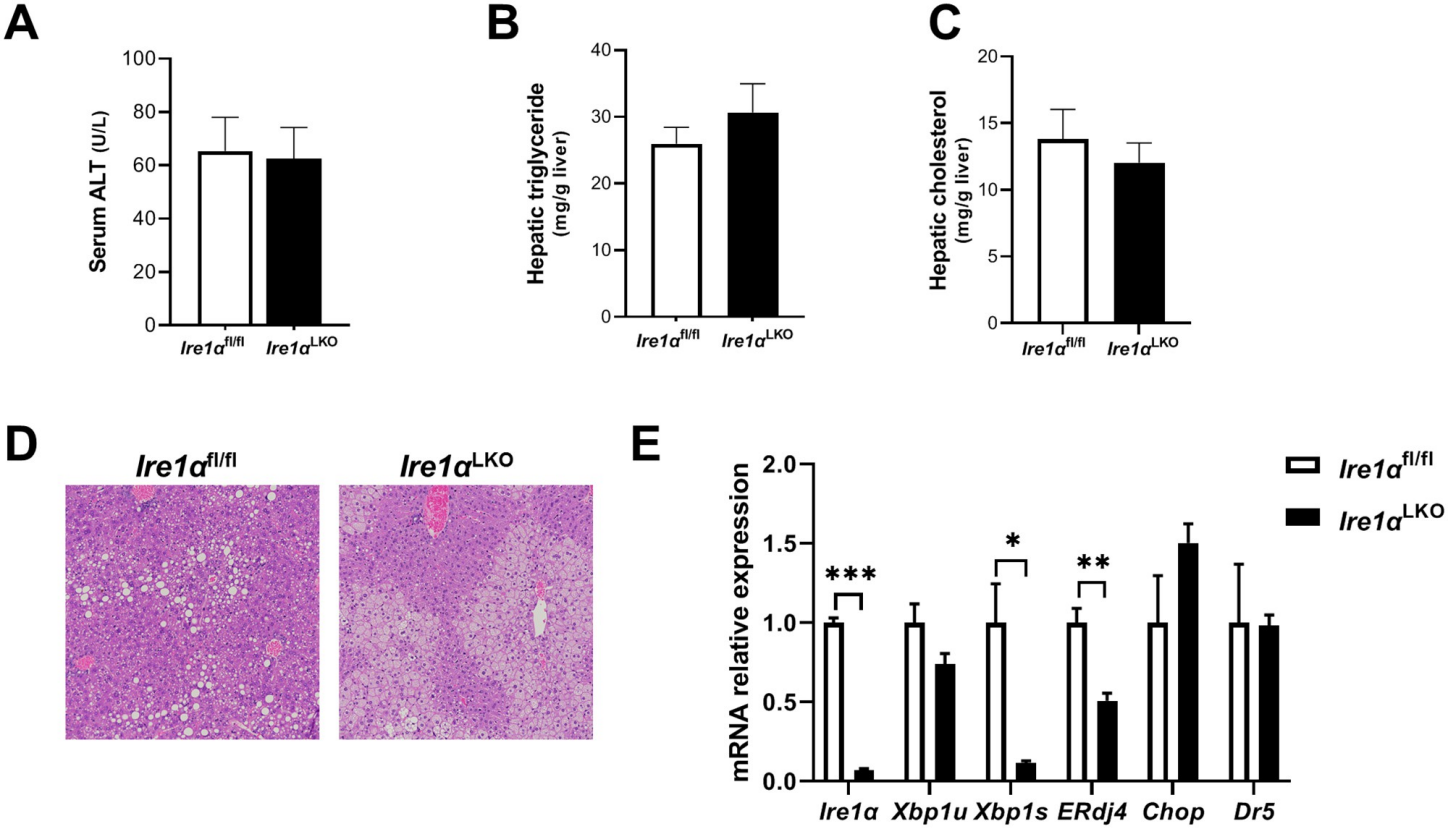
Serum ALT, hepatic triglyceride and cholesterol levels, and hepatic UPR gene expression in *Ire1α*^LKO^ and *Ire1α*^fl/fl^ mice fed HFS diet. *Ire1α*^LKO^ and *Ire1α*^fl/fl^ mice fed HFS diet for 5 weeks had similar (A) serum ALT, (B) hepatic triglyceride, and (C) hepatic cholesterol levels. (D) Representative images of H&E staining were shown. (E) Hepatic gene expression of *Ire1α*, *Xbp1s* and *ERdj4* were lower in *Ire1α*^LKO^ mice compared to *Ire1α*^fl/fl^ mice, while *Xbp1u* expression was similar. However, hepatic expression of the apoptotic genes (*Chop* and *Dr5*) was similar in both groups. n = 5 in each group. *p<0.05; **p<0.01, ***p<0.001 compared to *Ire1α*^fl/fl^ mice.

Hepatic XBP1 ablation causes enhanced expression and activity of its upstream activator IRE1α, therefore we investigated the effect of hepatic IRE1α deletion in the HFS dietary model of NASH. After HFS feeding, there was no difference in serum ALT, hepatic triglyceride and hepatic cholesterol levels between *Ire1α*^fl/fl^ and *Ire1α*^LKO^ mice (Fig 7A–7C). H&E staining demonstrates increased liver steatosis in both *Ire1α*^fl/fl^ and *Ire1α*^LKO^ mice after HFS feeding, but there was essentially no inflammation or ballooning (Fig 7D). As expected, hepatic gene expression of *Ire1α*, *Xbp1s* and the XBP1 downstream target *ERdj4*, but not XBP1 unspliced (*Xbp1u*), was decreased in *Ire1α*^LKO^ mice compared to *Ire1α*^fl/fl^ mice (p<0.001, p = 0.01, p = 0.002, respectively). The hepatic expression of the apoptotic genes *Chop* and *Dr5* was similar between HFS fed *Ire1α*^fl/fl^ and *Ire1α*^LKO^ mice (Fig 7E).

## Discussion

An increasing amount of data from both human and animal studies indicate that the hepatic UPR is important in the pathogenesis of NASH, as well as many other liver diseases. High fat, high caloric diets are a widely utilized murine model for obesity, hepatic steatosis and insulin resistance, however, we concomitantly administered dietary fructose/glucose since this can induce steatohepatitis with or without hepatic fibrosis. We have previously fed a high fat, high caloric diet with fructose/sucrose and demonstrated that *Xbp1*^LKO^ mice develop more rapid and severe steatohepatitis than *Xbp1*^*f*l/fl^ mice [4]. However, studies examining cell signaling in the liver have not adequately distinguished and determined the important roles of both hepatocytes and non-parenchymal cells. In whole liver homogenates, it is not possible to determine important cell signaling that occurs in hepatocytes, hepatic stellate cells and immune cells since strong signaling in one cell type can obscure important signaling pathways in other cell types. Therefore, we fed the HFS diet to *Xbp1*^LKO^ and *Xbp1*^fl/fl^ mice and isolated hepatocytes, hepatic stellate cells, macrophage and other immunologic cells to determine hepatocyte-specific changes of the UPR and other associated cell signaling pathways.

After confirming the cell purity and hepatocyte-specific deletion of XBP1 in these cell types, we performed RNA-Seq on hepatocytes isolated from *Xbp1*^LKO^ and *Xbp1*^fl/fl^ mice fed either chow or a HFS diet. GSEA pathway analysis on the hepatocyte RNA-Seq and whole liver RNA-Seq available from similarly treated mice revealed enhanced expression of stellate cell and bile duct pathways in the whole liver, but not hepatocytes. In line with increased gene expression of bile duct cell markers, we observed enhanced CK19 staining in *Xbp1*^LKO^ mice after HFS feeding compared to *Xbp1*^fl/fl^ mice. Ductular reaction has been associated with progressive NASH and liver fibrosis [12, 13]. The increased ductular reaction is consistent with greater liver injury and fibrosis in HFS-fed *Xbp1*^LKO^ mice compared to *Xbp1*^fl/fl^ mice. Furthermore, following HFS diet feeding, tissue and resident macrophage pathway expression differences also occurred, consistent with the enhanced steatohepatitis that occurred after HFS diet feeding.

*Xbp1*^LKO^ hepatocytes have markedly increased *Chop* gene expression, similar to our previous report in whole liver [4]. A major UPR regulator of CHOP is the PERK/eIF2α/ATF4 signaling cascade. PERK protein expression was seemingly paradoxically reduced in the *Xbp1*^LKO^ mice. This is consistent with previous report [11], although the etiology remains unclear. In addition, expression of the other ISR genes PKR, HRI and GCN2 was largely unchanged. Therefore, although p-eIF2α expression was increased with unchanged eIF2α in the *Xbp1*^LKO^ compared to *Xbp1*^fl/fl^ hepatocytes, it is unlikely that this is due to ISR kinase phosphorylation of eIF2α. Protein expression of the p-eIF2α target ATF4, its downstream target gene *Chop*, and the CHOP target gene *Dr5* was also increased in hepatocytes from *Xbp1*^LKO^ mice compared to *Xbp1*^fl/fl^ mice either under chow or HFS feeding. *Xbp1*^LKO^ mice have increases in hepatic p-IRE1α expression and resultant RIDD activation [9, 11], and it has been previously reported that CReP is a RIDD target [11]. In transfected cultured cells, IRE1α over-expression reduces CReP gene expression with a resultant increase in level of p-eIF2α expression. Therefore, the increased p-eIF2α expression may be due, at least in part, to the diminished CReP expression in hepatocytes isolated from *Xbp1*^LKO^ mice. In addition to increased pro-apoptotic *Chop* gene, hepatocyte expression of both pro-apoptotic proteins (BAX and BAK) and anti-apoptotic proteins (BCL-2 and BCL-XL) was significantly higher in chow fed *Xbp1*^LKO^ mice compared with *Xbp1*^fl/fl^ mice. These genotypic changes persisted after HFS diet. HFS feeding had no significant effects on expression of BAX, BAK and BCL-XL, but seemed to decrease BCL-2 expression.

In addition to CReP, we determined that several other RIDD targets were also diminished in *Xbp1*^LKO^ compared to *Xbp1*^fl/fl^ hepatocytes. Since *Xbp1*^LKO^ mice have increased IRE1α expression and RIDD activity, it raises the possibility that the phenotype of *Xbp1*^LKO^ mice may be due to increased RIDD activity. Therefore, we next fed the HFS diet to *Ire1α*^LKO^ mice, which have diminished hepatic expression of both IRE1α and XBP1. There was no difference in liver injury between the two genotypes, indicating that the phenotype of *Xbp1*^LKO^ mice is not merely due to the loss of hepatic XBP1 and that liver XBP1-independent signaling may also be important in the disease pathogenesis. It has been reported that hepatocyte specific *Ire1α*^-/-^ mice fed high-fat diet had significant microvesicular steatosis, whereas high fat diet-fed *Ire1α*^fl/fl^ mice developed macrovesicular steatosis [14], which is consistent with our findings. Of note, in our study, the *Xbp1*^LKO^ and *Xbp1*^fl/fl^ mice are in a C57BL/6 background strain, while the *Ire1α*^LKO^ and *Ire1α*^fl/fl^ mice are in a mixed C57BL/6/129 background strain, and the mechanism of Cre gene expression differed between these mice, so we cannot directly compare the results of the hepatocyte-specific IRE1α and XBP1 knockout genotypes. A previous report using *Xbp1*^LKO^ mice revealed liver UPR activation of several proteins and genes with fructose feeding, an effect that may be mediated via RIDD [15].

We identified gene expression of hepatocyte inflammatory pathways using RNA-Seq. The differentially expressed inflammatory pathways from chow fed *Xbp1*^LKO^ versus *Xbp1*^fl/fl^ hepatocytes were restricted to interferon alpha and gamma pathways. In contrast, following HFS diet feeding, *Xbp1*^LKO^ hepatocytes showed enrichment for inflammatory pathways including the TNFA_Signaling_Via_NFκB, Inflammatory_Response, and Apoptosis pathways. These findings contrast with findings on flow cytometry that showed no difference in immune cell numbers between genotypes fed the HFS diet. These data suggest that hepatocyte-driven inflammatory signaling may play a critical role in the immune injury in *Xbp1*^LKO^ mice. However, we cannot exclude the possibility that changes in smaller immune cell subsets not defined by canonical cell surface markers may contribute to the transcriptional activation of inflammatory pathways in the setting of HFS diet. For example, myeloid cell diversity identified by single-cell sequencing in NASH found distinct cell populations associated with specific environmental niches [16]. Lastly, we cannot exclude the possibility that changes in immune cell numbers in response to HFS feeding may occur at other time points.

The complex metabolic network regulating macrophage polarization has been previously described [17, 18]. However, a role for XBP1 in these networks has not been identified, despite known associations between the UPR and innate inflammation [19]. We observed increased numbers of immune cell subsets in chow-fed *Xbp1*^LKO^ compared to *Xbp1*^fl/fl^ mice including monocyte-derived Ly6c+ and Ly6c- macrophages, dendritic cells, and adaptive immune cells. This observation suggests that an aberrant hepatocyte UPR with potentially resultant unresolved ER stress can induce greater recruitment of immune cells to the liver at baseline, despite the lack of histologic evidence of hepatic inflammation [4]. Transcriptional profiling identified greater expression of immune genes in *Xbp1*^LKO^ mice fed HFS diet despite lower immune cell numbers, suggesting that changes in immune cell polarization may drive hepatic inflammation in these mice.

The gene expression of *Col1a1* and *Acta2* and protein expression of smooth muscle α actin were markedly increased in the hepatic stellate cells isolated from HFS diet fed *Xbp1*^LKO^ mice, compared to the other groups. This activation of hepatic stellate cells in HFS fed *Xbp1*^LKO^ mice is consistent with our previous report of enhanced liver fibrosis in *Xbp1*^LKO^ mice as early as 4 weeks after HFS feeding [4], as hepatic stellate cells are the major source of fibrogenesis. As expected, hepatic stellate cell *Xbp1* gene expression was not reduced by the hepatocyte-specific deletion of *Xbp1*. Therefore, the hepatic stellate cell activation is likely due to inflammatory signaling from hepatocytes, macrophages or other non-parenchymal cells. All the mice used in our study were males. It is unknown whether female liver specific XBP1 knockout mice will have similar findings.

In summary, this study analyzed the differential roles and signaling of different liver cell populations in the pathogenesis of dietary model of NASH. We consistently showed that loss of XBP1 in hepatocytes increased apoptotic pathway expression and inflammatory signaling in mice after HFS feeding. Hepatic stellate cells were activated in HFS fed *Xbp1*^LKO^ mice, but this appears to be predominantly secondary to signaling from hepatocytes, nonparenchymal cells or the increased baseline immune cell recruitment. We believe that a better understanding of the cell-specific UPR and inflammatory signaling in the liver in the pathogenesis of NASH may identify new targets for medical therapies.

## Supporting information

S1 Fig Gene set enrichment analysis of cell type signatures in isolated hepatocytes and whole liver from *Xbp1*^LKO^ and *Xbp1*^fl/fl^ mice fed chow or HFS diet. Gene set enrichment analysis (GSEA) using C8 collection of gene sets was performed on RNA-Seq data of hepatocytes isolated from *Xbp1*^LKO^ and *Xbp1*^fl/fl^ mice fed either chow or HFS diet. Same GSEA analysis was also performed with whole liver RNA-Seq data from similarly treated mice (GSE64824). The top 20 most enriched gene sets were shown comparing (A) chow-fed *Xbp1*^LKO^ whole liver to chow-fed *Xbp1*^fl/fl^ whole liver; (B) chow-fed *Xbp1*^LKO^ hepatocytes to chow-fed *Xbp1*^fl/fl^ hepatocytes; (C) HFS-fed *Xbp1*^LKO^ whole liver to HFS-fed *Xbp1*^fl/fl^ whole liver; and (D) HFS-fed *Xbp1*^LKO^ hepatocytes to HFS-fed *Xbp1*^fl/fl^ hepatocytes.
(TIF)

S2 Fig Ductular reaction was increased in *Xbp1*^LKO^ mice compared to *Xbp1*^fl/fl^ mice after HFS diet feeding. *Xbp1*^LKO^ and *Xbp1*^fl/fl^ mice were fed chow or HFS diet for 4 weeks (n = 4 in each group). Representative CK19 staining images were shown.
(TIF)

S3 Fig Flow cytometry gating strategy. Flow cytometry gating strategy identified eosinophils and neutrophils, B and T cells, dendritic cells, Ly6c+ and Ly6c- monocyte-derived macrophages (Mo-M), and CD64^high^ macrophages (M⏀).
(TIF)

S4 Fig Immune genes that were differentially expressed in whole liver but not isolated hepatocytes in *Xbp1*^LKO^ and *Xbp1*^fl/fl^ mice fed chow or HFS diet. RNA-Seq was performed on hepatocytes isolated from *Xbp1*^LKO^ and *Xbp1*^fl/fl^ mice fed either chow or HFS diet and was compared to whole liver RNA-Seq data from similarly treated mice (GSE64824). Heatmaps demonstrated immune genes that were differentially expressed in whole liver, but not isolated hepatocytes of mice fed (A) chow but not HFS diet, (B) HFS diet, but not chow and (C) either diet.
(TIF)

S1 Raw images Raw images of all the blots. (PDF)

S1 File Antibodies used in western blotting. (DOCX)

S2 File Antibodies used in flow cytometry. (DOCX)
