# Supplementary figures and images for: Mechanisms of liver injury in high fat sugar diet fed mice that lack hepatocyte X-box binding protein 1
Source: PLoS One. 2022 Jan 14;17(1):e0261789. doi: 10.1371/journal.pone.0261789 (PMC8759640; doi:10.1371/journal.pone.0261789)

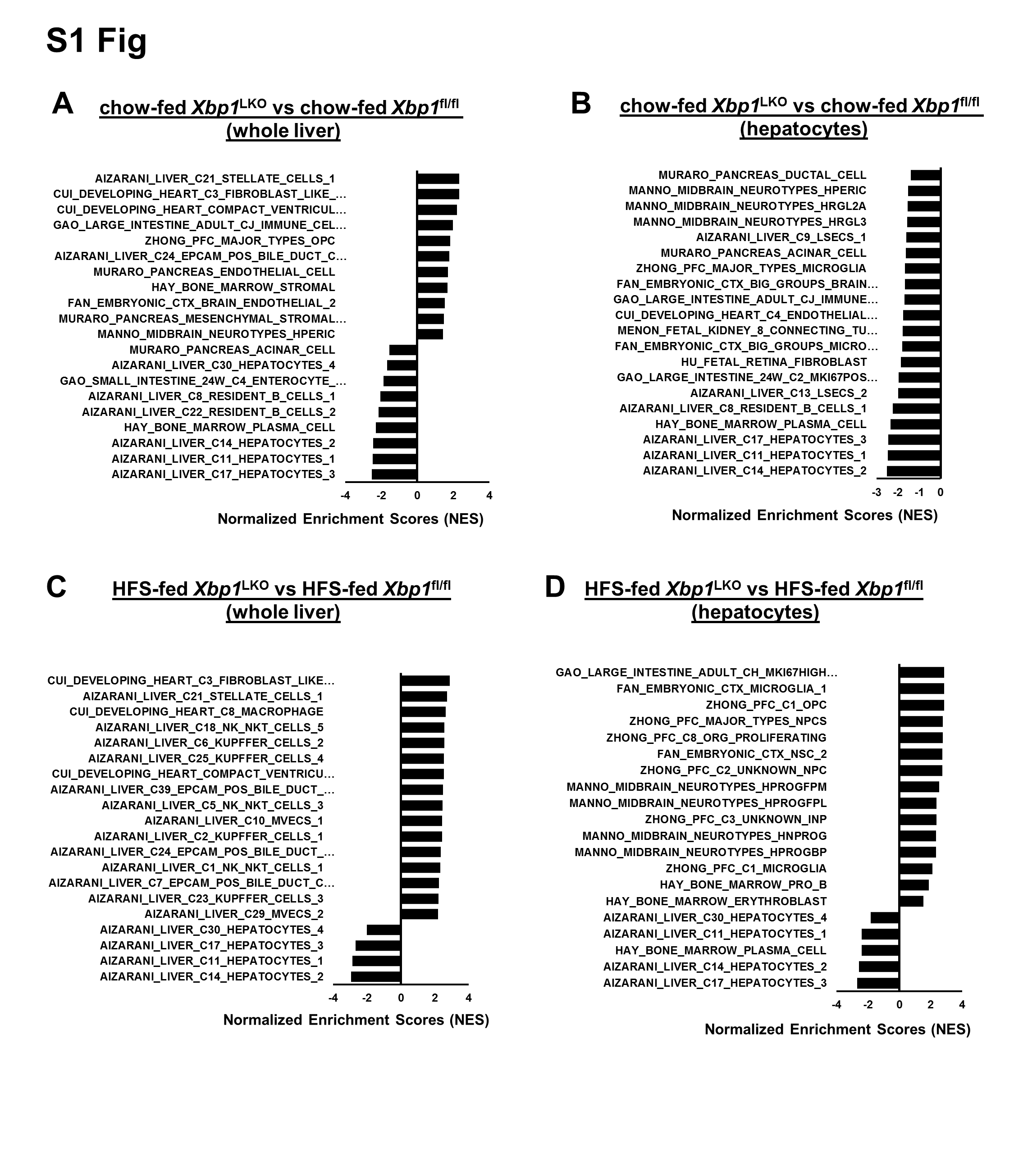

Supplement: S1 Fig — Gene set enrichment analysis (GSEA) using C8 collection of gene sets was performed on RNA-Seq data of hepatocytes isolated from Xbp1LKO and Xbp1fl/fl mice fed either chow or HFS diet. Same GSEA analysis was also performed with whole liver RNA-Seq data from similarly treated mice (GSE64824). The top 20 most enriched gene sets were shown comparing (A) chow-fed Xbp1LKO whole liver to chow-fed Xbp1fl/fl whole liver; (B) chow-fed Xbp1LKO hepatocytes to chow-fed Xbp1fl/fl hepatocytes; (C) HFS-fed Xbp1LKO whole liver to HFS-fed Xbp1fl/fl whole liver; and (D) HFS-fed Xbp1LKO hepatocytes to HFS-fed Xbp1fl/fl hepatocytes. (TIF) [file pone.0261789.s001.TIF]

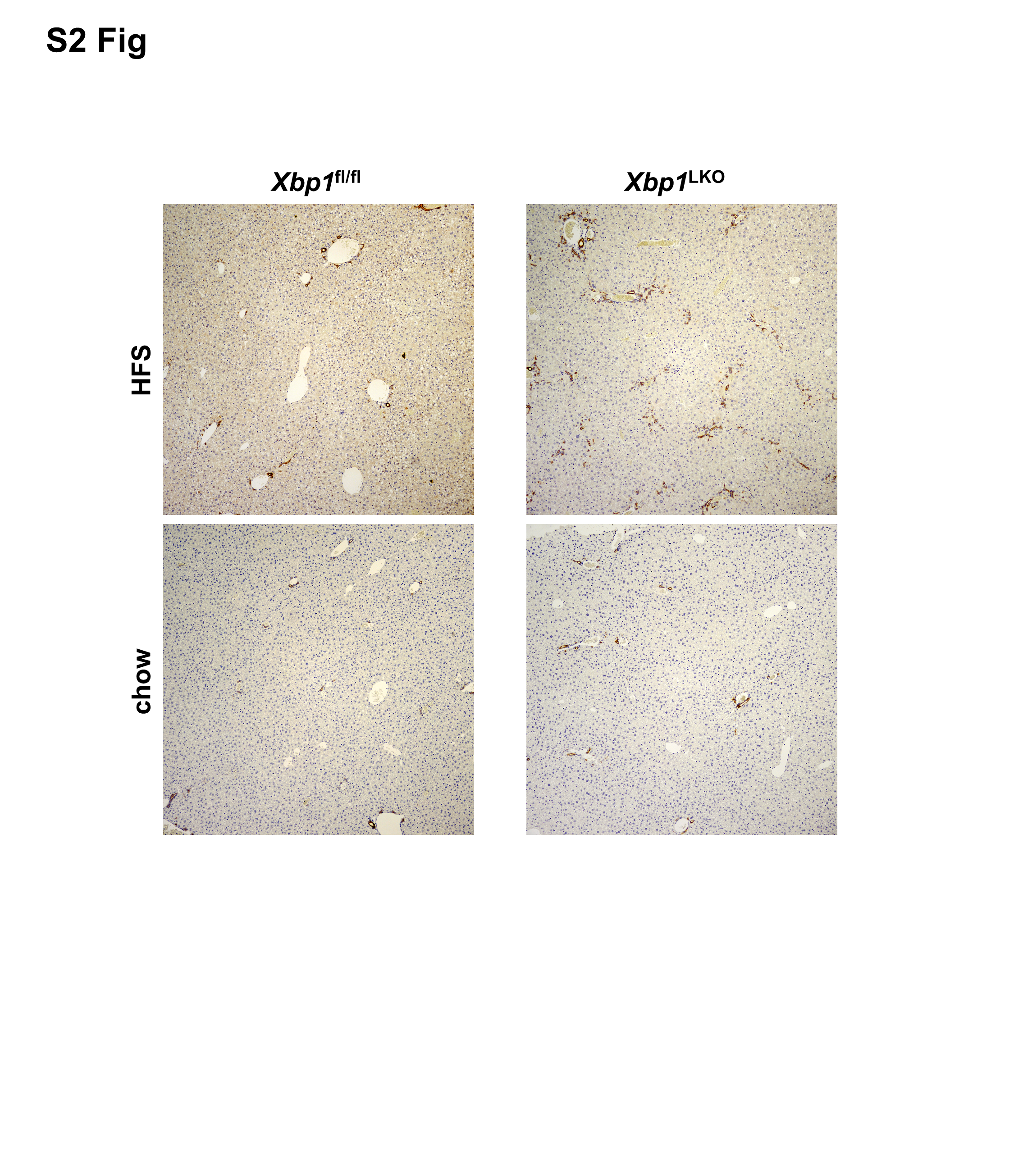

Supplement: S2 Fig — Xbp1LKO and Xbp1fl/fl mice were fed chow or HFS diet for 4 weeks (n = 4 in each group). Representative CK19 staining images were shown. (TIF) [file pone.0261789.s002.tif]

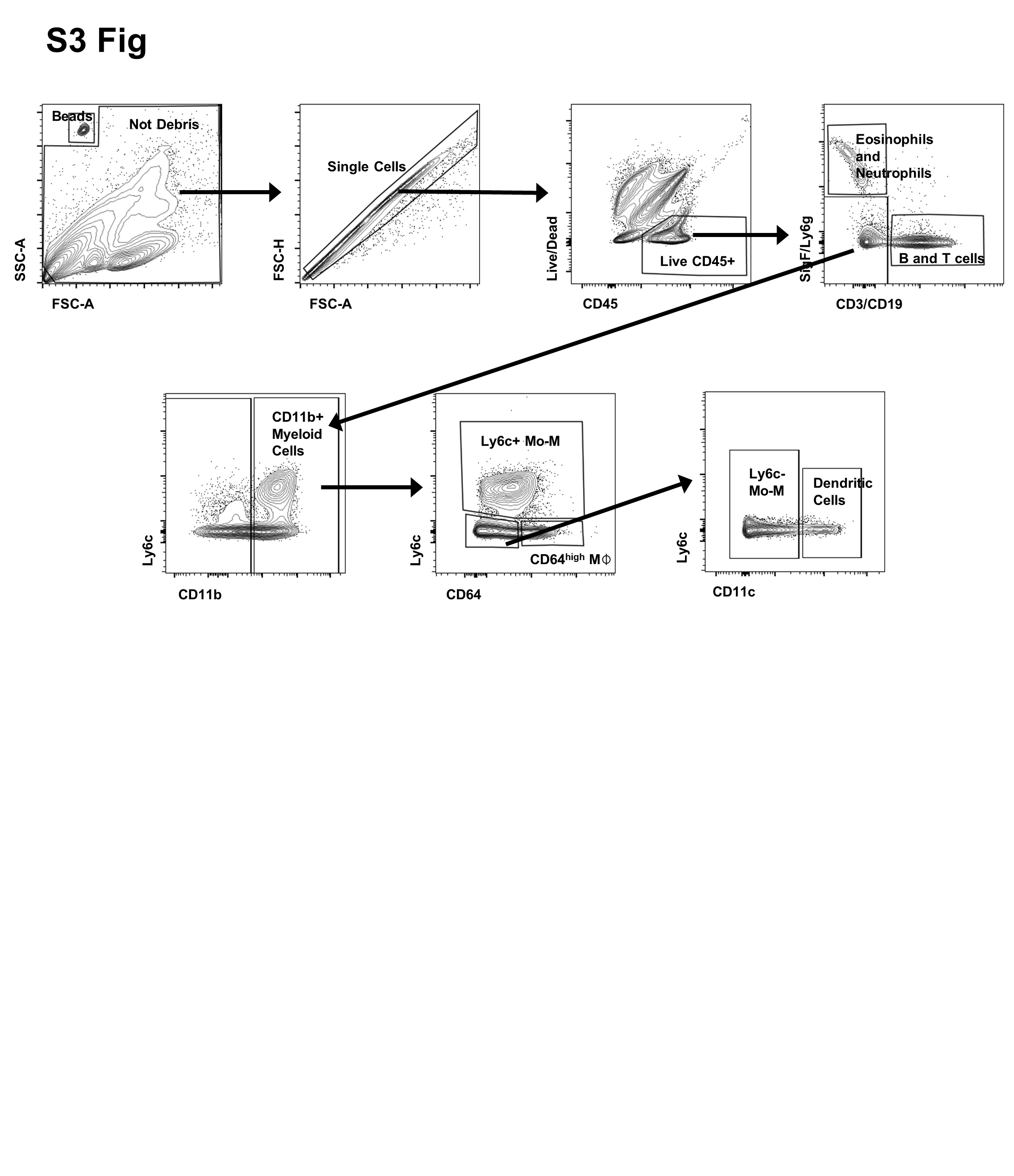

Supplement: S3 Fig — Flow cytometry gating strategy identified eosinophils and neutrophils, B and T cells, dendritic cells, Ly6c+ and Ly6c- monocyte-derived macrophages (Mo-M), and CD64high macrophages (M⏀). (TIF) [file pone.0261789.s003.tif]

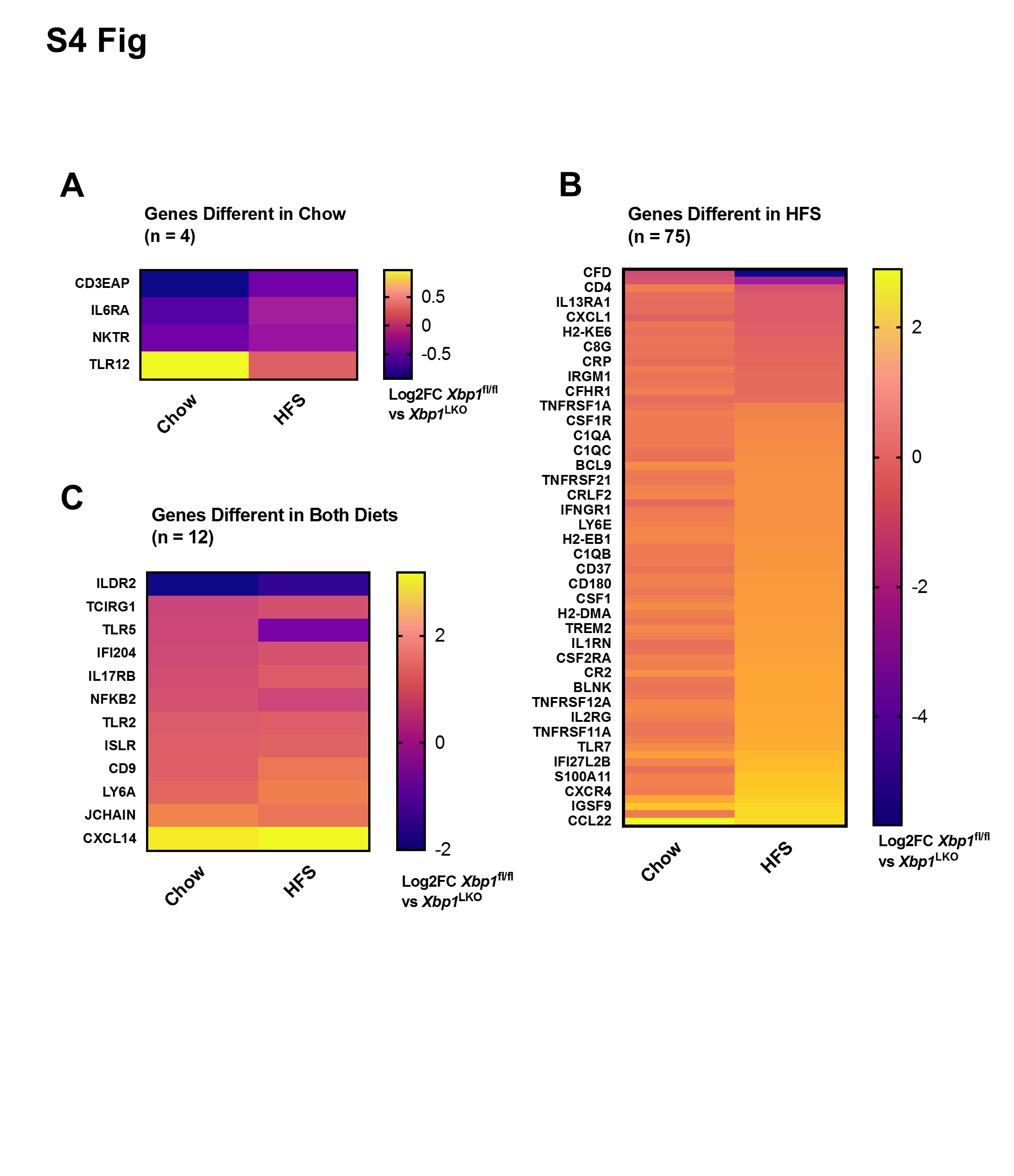

Supplement: S4 Fig — RNA-Seq was performed on hepatocytes isolated from Xbp1LKO and Xbp1fl/fl mice fed either chow or HFS diet and was compared to whole liver RNA-Seq data from similarly treated mice (GSE64824). Heatmaps demonstrated immune genes that were differentially expressed in whole liver, but not isolated hepatocytes of mice fed (A) chow but not HFS diet, (B) HFS diet, but not chow and (C) either diet. (TIF) [file pone.0261789.s004.tif]
