# Supplementary material for: Mechanisms of liver injury in high fat sugar diet fed mice that lack hepatocyte X-box binding protein 1
Source: PLoS One. 2022 Jan 14;17(1):e0261789. doi: 10.1371/journal.pone.0261789 (PMC8759640; doi:10.1371/journal.pone.0261789)

Figure 1D
ECL

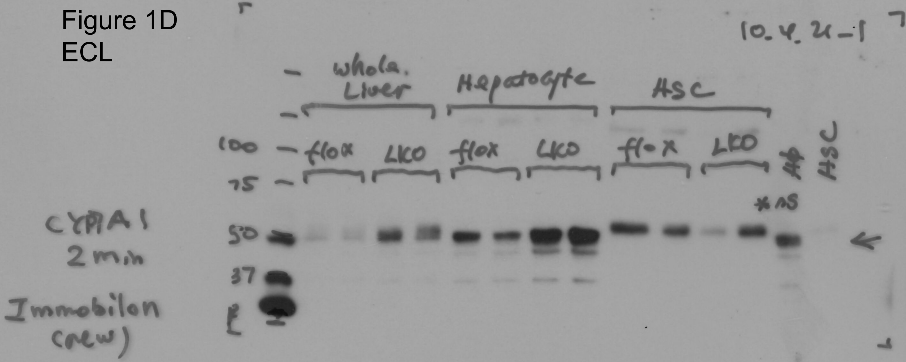

Figure 1D
ECL

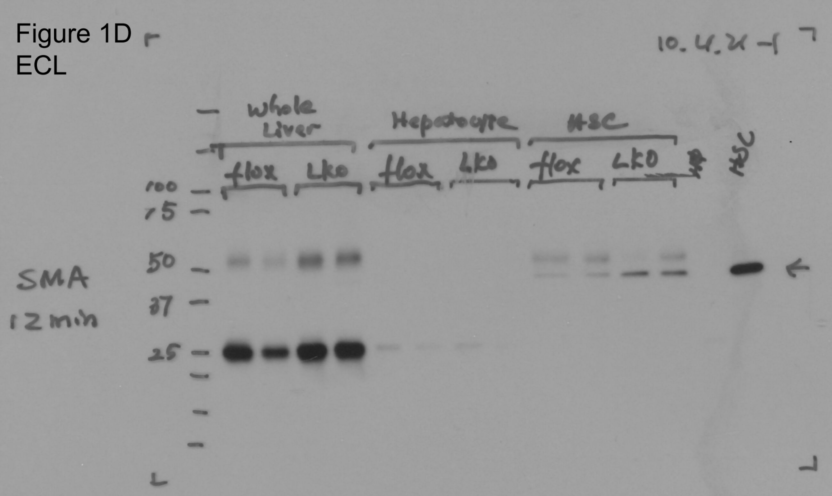

Figure 1D
ECL

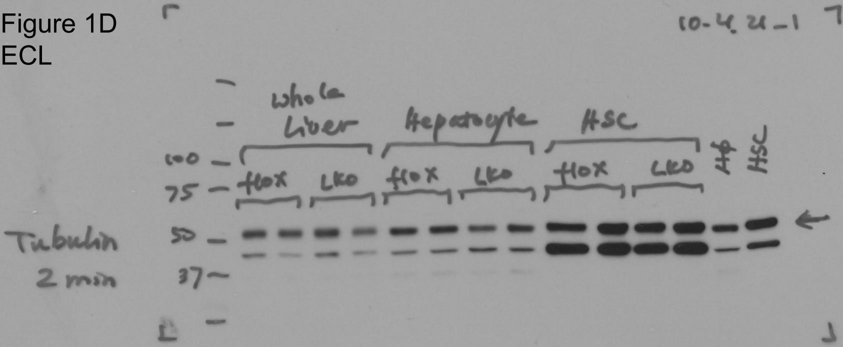

Fig 3C

ECL

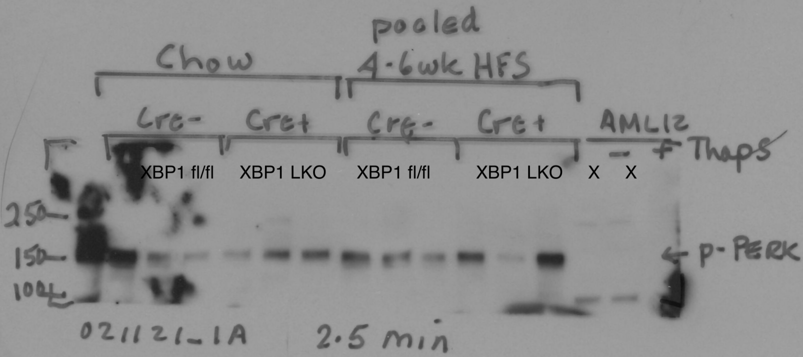
pooled
Chow
4-6wk HFS
Cre-
Cre+
Cre-
Cre+
AML12
- + Thaps
XBP1 fl/fl
XBP1 LKO
XBP1 fl/fl
XBP1 LKO
X
X
250
150
100
p-PERK
021121-1A
2.5 min

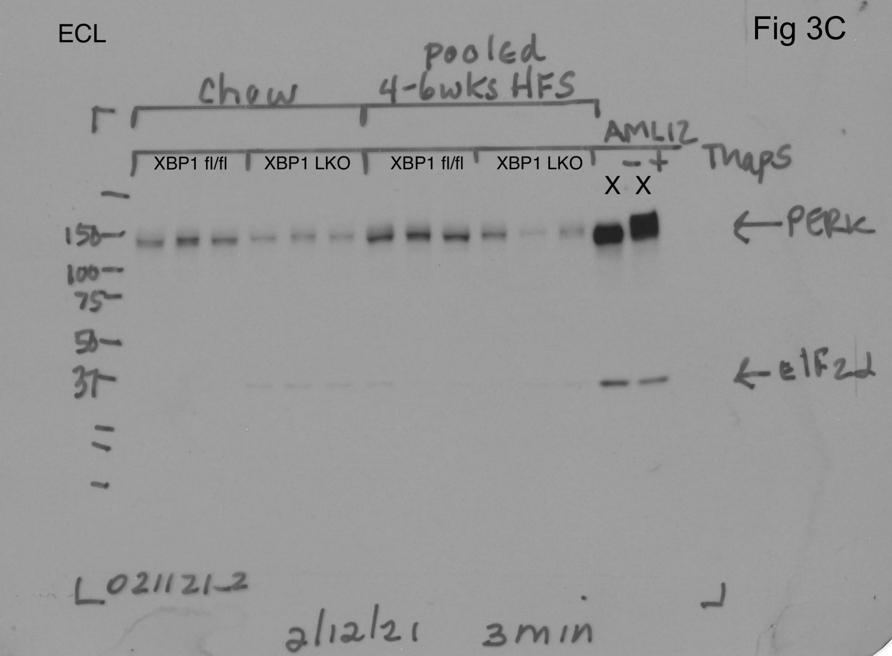

ECL
Fig 3C
pooled
Chow
4-6wks HFS
AML12
XBP1 fl/fl
XBP1 LKO
XBP1 fl/fl
XBP1 LKO
- +
Thaps
X X
150
100
75
50
37
←PERK
←eIF2α
L021121-2
2/12/21 3min

Fig 3C

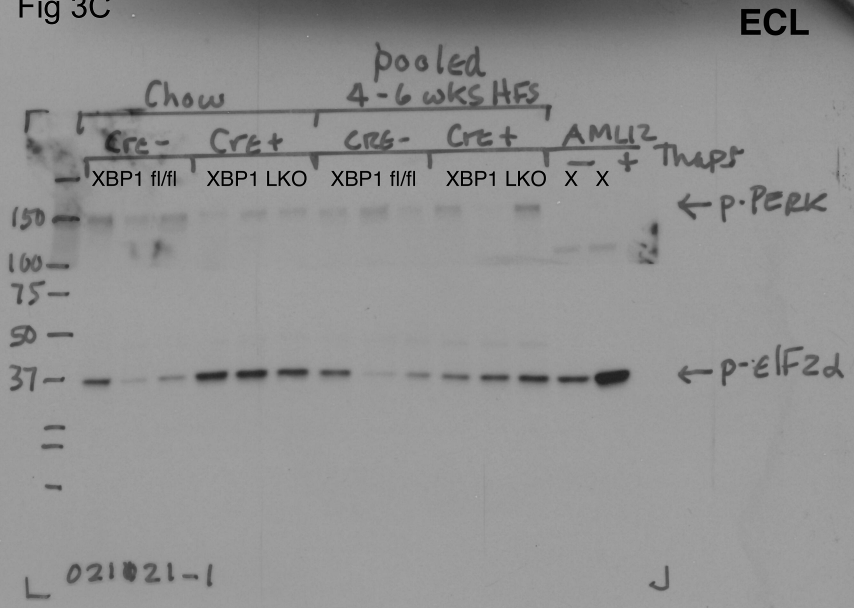
ECL
pooled
Chow
4-6 wks HFS
Cre -
Cre +
CRE -
Cre +
AML12
- + Thaps
XBP1 fl/fl
XBP1 LKO
XBP1 fl/fl
XBP1 LKO
X
X
← p-PERK
← p-eIF2α
150
100
75
50
37
021021-1

Fig 3C

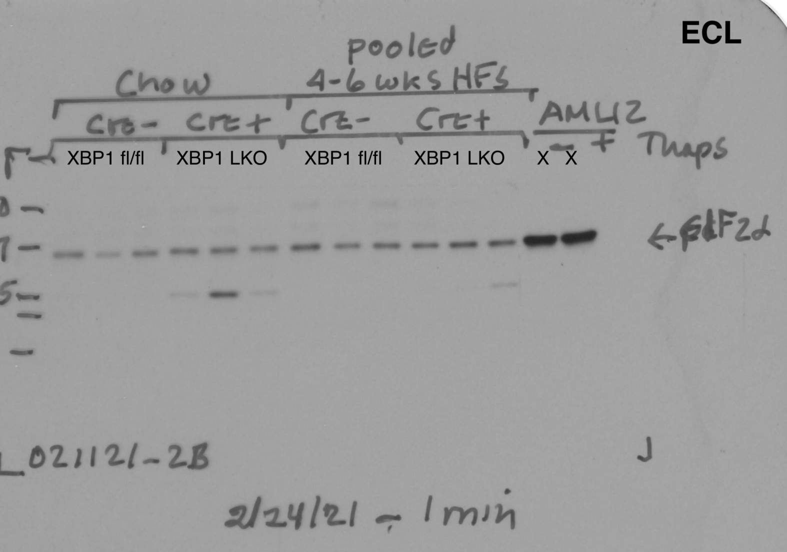
ECL
pooled
Chow
4-6 wks HFS
Cre- Cre+ Cre- Cre+
AML12
- + Thaps
XBP1 fl/fl
XBP1 LKO
XBP1 fl/fl
XBP1 LKO
X X
50
37
25
← eIF2α
L021121-2B
2/24/21 - 1min

Fig 3C

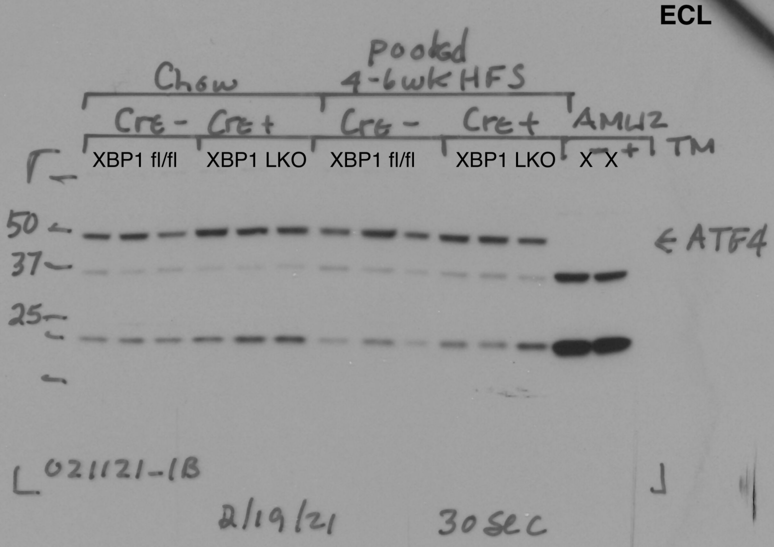
ECL
Pooled
Chow
4-6wk HFS
Cre - Cre +
Cre - Cre +
AM12
XBP1 fl/fl
XBP1 LKO
XBP1 fl/fl
XBP1 LKO
X X
- +
TM
50
37
25
← ATF4
021121-1B
2/19/21
30sec

Fig 4

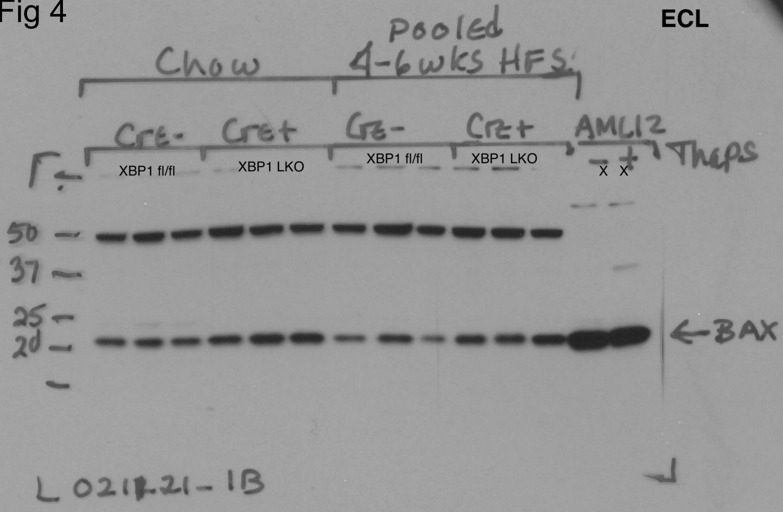
ECL
Pooled
Chow
4-6wks HFS
Cre- Cre+ Cre- Cre+ AML12
XBP1 fl/fl
XBP1 LKO
XBP1 fl/fl
XBP1 LKO
- +
X X
Thaps
50
37
25
20
← BAX
L 021421-1B

Fig 4

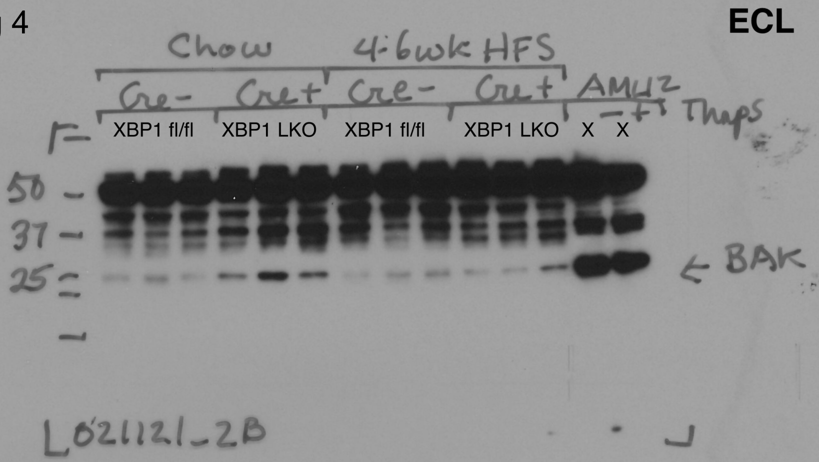
ECL
Chow
4-6wk HFS
Cre-
Cre+
Cre-
Cre+
AM12
XBP1 fl/fl
XBP1 LKO
XBP1 fl/fl
XBP1 LKO
- +
Thaps
X
X
50
37
25
BAK
L021121_2B

Fig 4

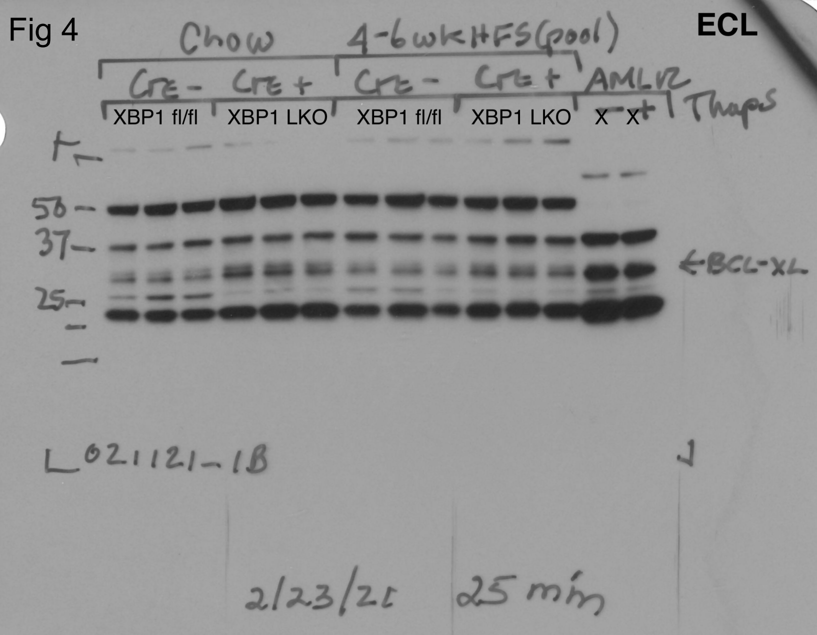
ECL
Chow
4-6wk HFS (pool)
Cre- Cre+ Cre- Cre+
AML12
- +
Thaps
XBP1 fl/fl
XBP1 LKO
XBP1 fl/fl
XBP1 LKO
X X
50
37
25
←BCL-XL
L021121-1B
2/23/21
25 min

Fig 4

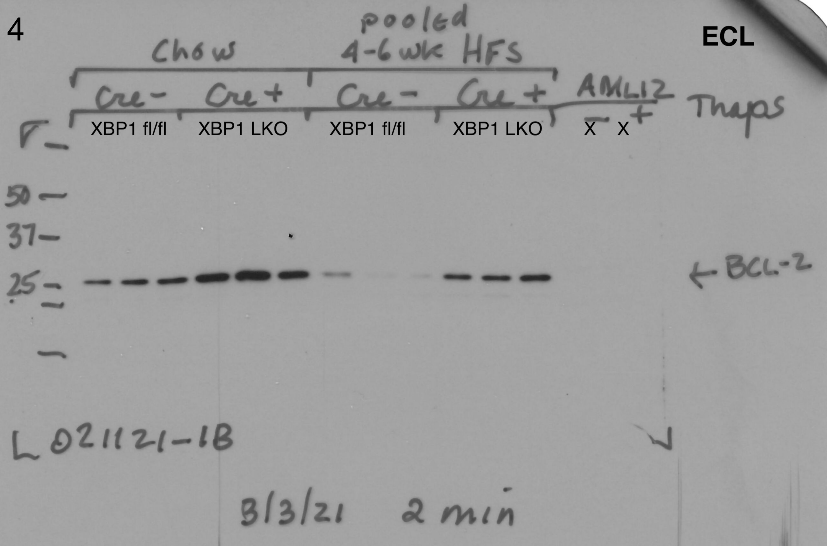

ECL
pooled
4-6 wk HFS
Chow
Cre- Cre+ Cre- Cre+
AML12
Thaps
XBP1 fl/fl
XBP1 LKO
XBP1 fl/fl
XBP1 LKO
X - X +
50
37
25
BCL-2
L 021121-1B
3/3/21 2 min

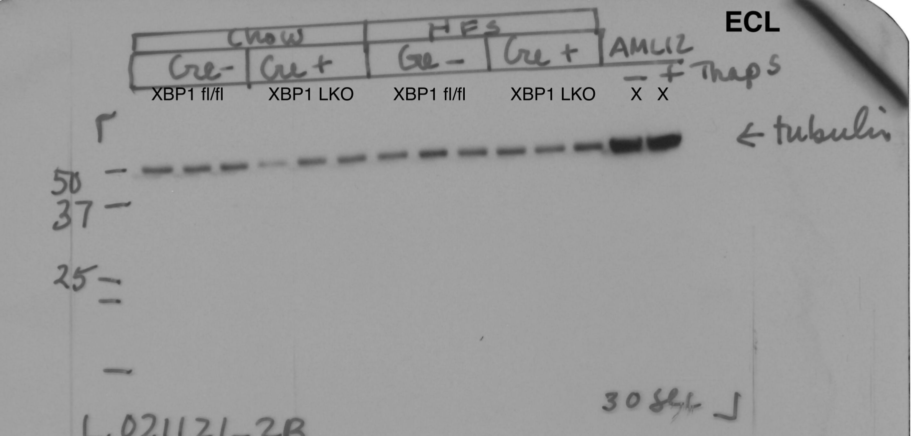

Fig 3C and Fig 4

Fig 5C
ECL

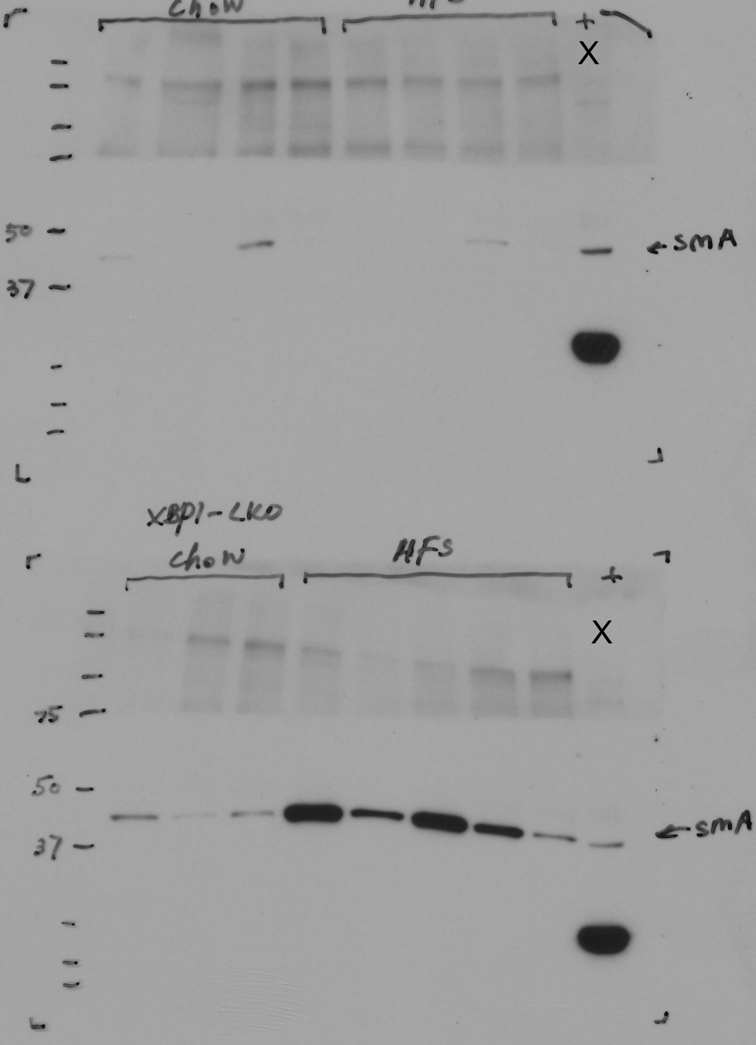

Figure 5C
ECL

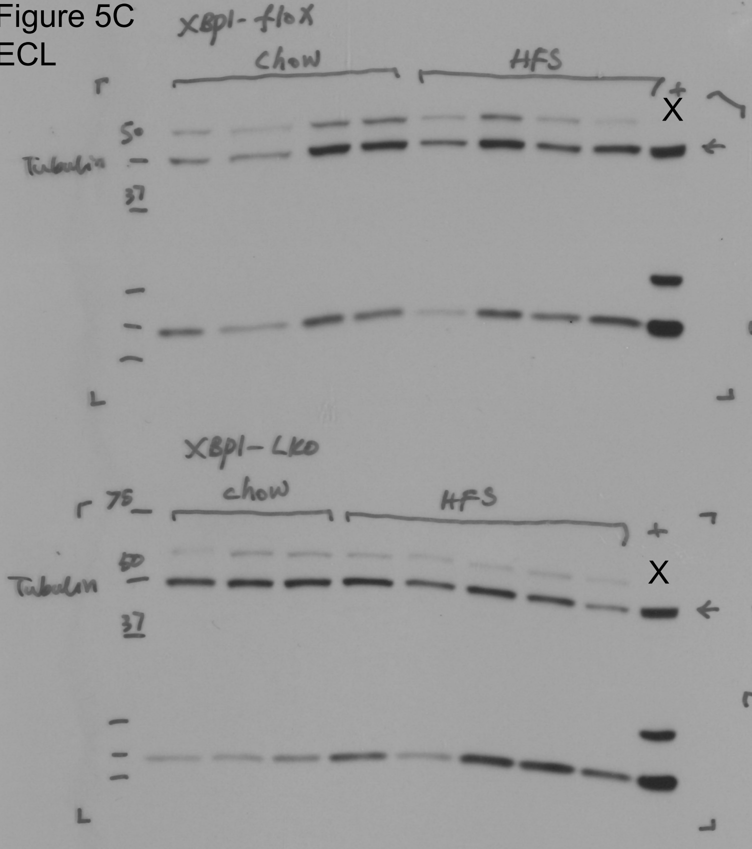

Supplement: S1 Raw images — (PDF) [file pone.0261789.s005.pdf]
