# Supplementary material for: Mechanisms of liver injury in high fat sugar diet fed mice that lack hepatocyte X-box binding protein 1
Source: PLoS One. 2022 Jan 14;17(1):e0261789. doi: 10.1371/journal.pone.0261789 (PMC8759640; doi:10.1371/journal.pone.0261789)
Supplement: S1 File — (DOCX) [file pone.0261789.s006.docx]

| **Antibody** | **Vendor** | **Catalog Number** | **Host species** | **Type** | **Antigen** | **Dilution** |
| --- | --- | --- | --- | --- | --- | --- |
| alpha-Tubulin | Proteintech | 11224-1-AP | Rabbit | polyclonal | Alpha Tubulin fusion protein | 1:5000 |
| Bax | Cell Signaling | 2772 | Rabbit | polyclonal | amino-terminal residues of human Bax | 1:1000 |
| Bak | Cell Signaling | 3814 | Rabbit | polyclonal | residues surrounding Gly82 of human Bak | 1:1000 |
| Bcl-2 | Cell Signaling | 2876 | Rabbit | polyclonal | a synthetic peptide corresponding to the carboxy-terminus of Bcl-2 alpha | 1:1000 |
| Bcl-XL | Cell Signaling | 2764 | Rabbit | monoclonal | residues surrounding Asp61 of human Bcl-xL | 1:1000 |
| ATF4 | Cell Signaling | 11815 | Rabbit | monoclonal | residues near the carboxy terminus of human ATF-4 | 1:1000 |
| eIF2α | Cell Signaling | 9722 | Rabbit | polyclonal | the carboxy-terminal sequence of eIF2α | 1:1000 |
| PERK | Cell Signaling | 3192 | Rabbit | monoclonal | a synthetic peptide corresponding to the sequence of human PERK | 1:1000 |
| phospho-eIF2alpha (ser51) | Cell Signaling | 9721 | Rabbit | polyclonal | detects endogenous eIF2alpha only when phosphorylated at Ser51 | 1:1000 |
| phospho-PERK (Thr980) | Thermo Fisher | MA5-15033 | Rabbit | monoclonal | Synthetic phosphopeptide corresponding to residues surrounding pThr980 of mouse PERK | 1:500 |
| Smooth muscle α actin | Sigma | A5228 | Mouse | monoclonal | N-terminal synthetic decapeptide of α-smooth muscle actin | 1:2000 |
| CYP7A1 | Proteintech | 18054-1-AP | Rabbit | polyclonal | CYP7A1 fusion protein | 1:1000 |
