# Supplementary material for: Mechanisms of liver injury in high fat sugar diet fed mice that lack hepatocyte X-box binding protein 1
Source: PLoS One. 2022 Jan 14;17(1):e0261789. doi: 10.1371/journal.pone.0261789 (PMC8759640; doi:10.1371/journal.pone.0261789)
Supplement: S2 File — (DOCX) [file pone.0261789.s007.docx]

| **Antibody** | **Manufacturer** | **Fluorochrome** | **Host** | **Clone** | **Concentration per 5 million cells** |
| --- | --- | --- | --- | --- | --- |
| Anti-Ly6c | BD Biosciences | BV421 | Rat monoclonal | AL-21 | 0.02 |
| Anti-CD45 | BD Biosciences | FITC | Rat monoclonal | 30-F11 | 0.005 |
| Anti-CD64 | BD Biosciences | PE | Mouse monoclonal | X54-5/7.1 | 0.5 |
| Anti-Siglec-F | BD Biosciences | PE-CF594 | Rat monoclonal | E50-2440 | 0.7 |
| Anti-Ly6g | BD Biosciences | PE-CF594 | Rat monoclonal | 1A8 | 0.03 |
| Anti-CD11c | BD Biosciences | APC | Armenian Hamster monoclonal | N418 | 0.06 |
| Anti-CD11b | BioLegend | APC-Cy7 | Rat monoclonal | M1/70 | 0.004 |
| Anti-CD3 | BD Biosciences | AF 700 | Hamster monoclonal | 500A2 | 0.7 |
| Anti-CD19 | BD Biosciences | AF 700 | Rat monoclonal | 1D3 | 1.0 |
